# TSPY1 suppresses USP7-mediated p53 function and promotes spermatogonial proliferation

**DOI:** 10.1038/s41419-018-0589-7

**Published:** 2018-05-10

**Authors:** Ying Shen, Wenling Tu, Yunqiang Liu, Xiling Yang, Qiang Dong, Bo Yang, Jinyan Xu, Yuanlong Yan, Xue Pei, Mohan Liu, Wenming Xu, Yuan Yang

**Affiliations:** 1 Department of Medical Genetics, State Key Laboratory of Biotherapy, West China Hospital, Sichuan University and Collaborative Innovation Center, Chengdu, 610041 China; 2 Joint Laboratory of Reproductive Medicine, SCU-CUHK, Key Laboratory of Obstetric, Gynecologic and Pediatric Diseases and Birth Defects of Ministry of Education, West China Second University Hospital, Sichuan University, Chengdu, 610041 China; 3 Department of Urology, West China Hospital, Sichuan University, Chengdu, 610041 China

## Abstract

Testis-specific protein Y-linked 1 (TSPY1) is expressed predominantly in adult human spermatogonia and functions in the process of spermatogenesis; however, our understanding of the underlying mechanism is limited. Here we observed that TSPY1, as an interacting partner of TSPY-like 5 (TSPYL5), enhanced the competitive binding of TSPYL5 to ubiquitin-specific peptidase 7 (USP7) in conjunction with p53. This activity, together with its promotion of TSPYL5 expression by acting as a transcription factor, resulted in increased p53 ubiquitylation. Moreover, TSPY1 could decrease the p53 level by inducing the degradation of ubiquitinated USP7. We demonstrated that the promotion of p53 degradation by TSPY1 influenced the activity of p53 target molecules (CDK1, p21, and BAX) to expedite the G2/M phase transition and decrease cell apoptosis, accelerating cell proliferation. Taken together, the observations reveal the significance of TSPY1 as a suppressor of USP7-mediated p53 function in inhibiting p53-dependent cell proliferation arrest. By simulating TSPY1 function in Tspy1-deficient spermatogonia derived from mouse testes, we found that TSPY1 could promote spermatogonial proliferation by decreasing the Usp7-modulated p53 level. The findings suggest an additional mechanism underlying the regulation of spermatogonial p53 function, indicating the significance of TSPY1 in germline homeostasis maintenance and the potential of TSPY1 in regulating human spermatogonial proliferation via the USP7-mediated p53 signaling pathway.

## Introduction

*TSPY1* (testis-specific protein Y-linked 1) is the highest-copy-number member of human multicopy genes^1–3^. Excessive amplification of *TSPY1* copies in the male-specific region of the Y chromosome (MSY) suggests that this gene may have a vital function in the physiological process of spermatogenesis^4,5^. Previous studies have shown that *TSPY1* can partially rescue spermatogenesis of KIT-deficient mice and the copy dosage of the *TSPY1* gene cluster has a significantly positive correlation with sperm production^6–8^, providing additional direct evidence of the involvement of *TSPY1* in human spermatogenesis.

Research is ongoing to investigate the molecular mechanisms of the functions of the cancer/testis protein encoded by the *TSPY1* gene^9,10^. Recent studies have shown that TSPY1 promotes cell proliferation by acting as an enhancer of the phosphorylation activity of cyclin B1-cyclin-dependent kinase 1 (CDK1) on histone H1 to accelerate the G2/M phase transition^11,12^. Additionally, TSPY1 increases protein synthesis and gene transcription by interacting with eukaryotic translation elongation factor 1A and activates numerous growth-related cellular functions by regulating the expression of endogenous androgen receptor-target genes^13,14^. However, our understanding of the mechanisms of TSPY1 functions in testis is significantly limited. Elucidation of these mechanisms is an important step in determining the role of this MSY-encoded protein in spermatogenesis and understanding the reason why *TSPY1* dosage deficiency confers an increased risk of spermatogenic failure and male infertility^7,8^.

Using a TSPY1-interacting partner (TSPY-like 5, TSPYL5) in adult human testicular tissue as an entry point, in this study, we investigated the functional pathway through which TSPY1 influences cell biological phenotypes in human somatic cells. A role of TSPY1 in the functional pathway for the modulation of Tspy1-deficient mouse spermatogonial proliferation was detected. With this work, we reveal that TSPY1 suppresses ubiquitin-specific peptidase 7 (USP7)-mediated p53 function; the promotion of spermatogonial proliferation by TSPY1 through the p53 signaling pathway probably contributes to human spermatogenesis.

## Results

### Screen for interacting proteins of TSPY1 in adult human testis

#### TSPYL5 is a focal interacting partner of TSPY1

TSPY1S, which contains 308 amino acids, is a major protein variant of TSPY1 in human testis^15^. With TSPY1S as bait, a total of 51 protein-encoding genes were screened in an adult human testicular cDNA library using the yeast two-hybrid system. We focused on the *TSPYL5* gene for the following reasons. (1) Among the three most frequent genes in the positive clones, it is the only gene expressed predominantly in adult human testis (http://www.proteinatlas.org; http://humanproteomemap.org). (2) The binding of TSPYL5 and TSPY1 may be functionally significant considering that TSPYL5 is an autosomal homologue of TSPY1 and that the functional structure of TSPY1 involves dimerization^13,16^. (3) TSPYL5-mediated promotion of cell proliferation has been reported^17,18^. For these reasons, we hypothesized that TSPY1 could be involved in the TSPYL5 functional pathway.

#### Validation of the physical interaction of TSPY1 with TSPYL5

**Fig. 1:**
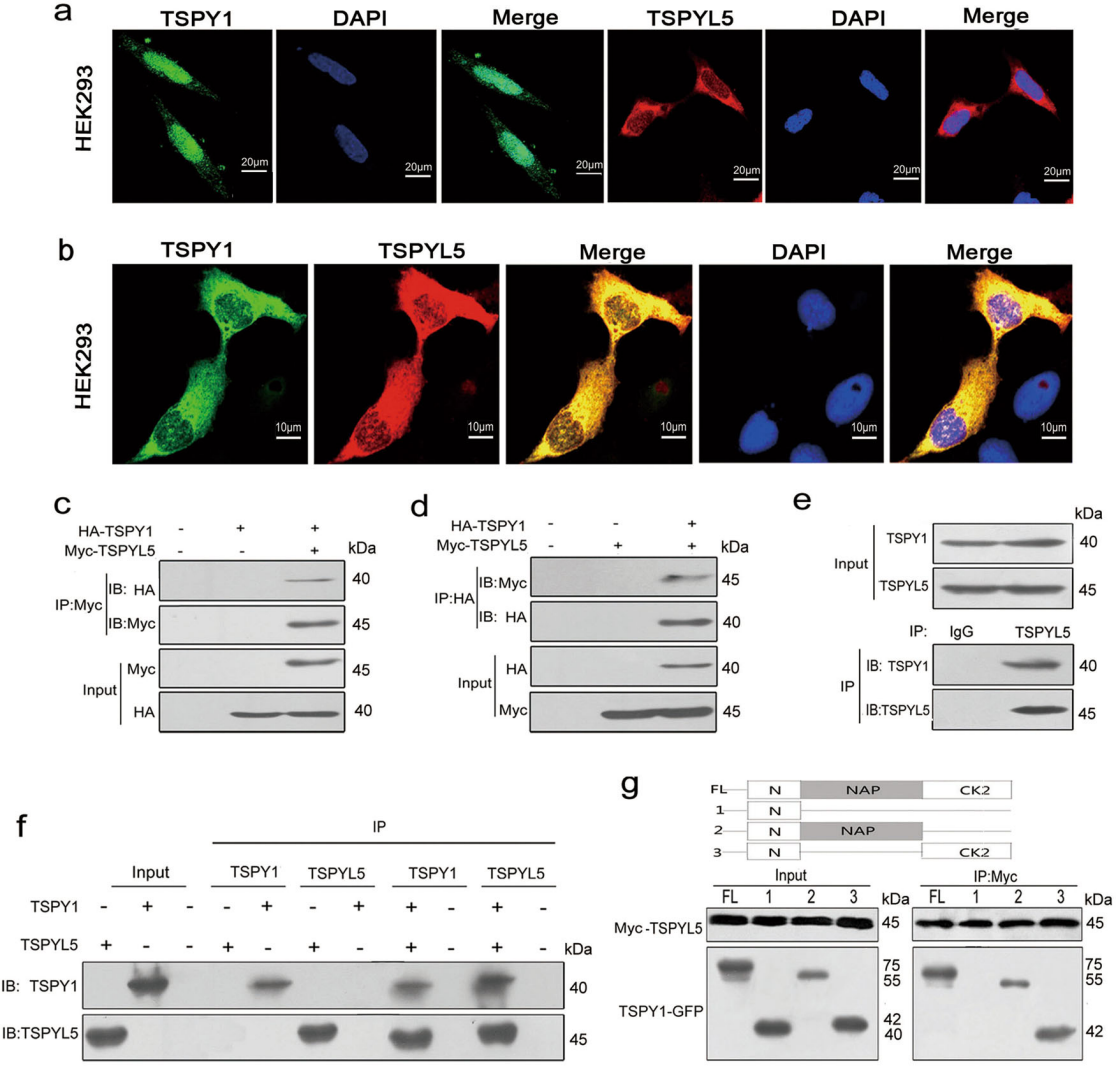
Cellular locations and physical interaction of TSPY1 and TSPYL5. **a** Immunofluorescence assays showed that TSPY1 (green) was mainly located in the nucleus, and TSPYL5 (red) was mainly present in the cytoplasm when the two proteins were individually expressed in HEK293 cells. Cells were counterstained with DAPI to label the nuclei (blue). The scale bar corresponds to 20 μm. **b** TSPY1 and TSPYL5 were primarily co-localized in the cytoplasm (yellow) when they were expressed together in HEK293 cells. The scale bar corresponds to 10 μm. **c**, **d** Co-IP assays showed that HA-TSPY1 and Myc-TSPYL5 could interact in HEK293 cells. **e** Co-IP assays showed the interaction between endogenous TSPY1 and TSPYL5 in A549 cells. **f** In vitro binding assays showed a direct interaction between TSPY1 and TSPYL5. **g** Schematic of the TSPY1 domains and the binding tests of the domains to TSPYL5. The Co-IP results showed that the NAP and CK2 domains of TSPY1 could bind to TSPYL5 in HEK293 cells

After confirming the binding of TSPY1 and TSPYL5 in AH109 yeast cells (Supplementary Fig. 1), they were individually transfected into TSPY1- and TSPYL5-null HEK293 cells (Supplementary Fig. 2). We observed that TSPY1 was mainly present in the nucleus, and TSPYL5 was mainly located in the cytoplasm (Fig. 1a). However, TSPY1 was mainly located in the cytoplasm when they were co-transfected (Fig. 1b). The similar cellular location of the two proteins was observed in HepG2, another TSPY1- and TSPYL5-null cell model (Supplementary Fig. 2, 3). For A549 cells expressing endogenous TSPY1 and TSPYL5 (Supplementary Fig. 2), the detection of nuclear and cytoplasmic extracts showed the co-location of these two proteins in the cytoplasm (Supplementary Fig. 4). Importantly, immunohistochemical detection of human testicular tissue sections showed that both proteins were abundantly expressed in spermatogonia with significant co-location in the cytoplasm (Supplementary Fig. 5). Further co-immunoprecipitation (Co-IP) analyses confirmed the binding between exogenous HA-TSPY1 and Myc-TSPYL5 in HEK293 cells (Fig. 1c, d) and between endogenous TSPY1 and TSPYL5 in A549 cells (Fig. 1e). In vitro binding assays suggested a direct interaction between TSPY1 and TSPYL5 (Fig. 1f). In addition, our results indicated that both the NAP and C-terminal casein kinase II (CK2) domains of TSPY1 could bind to TSPYL5 (Fig. 1g). Taken together, these observations provided convincing evidence of the physical binding of TSPY1 with TSPYL5.

### Influence of the interaction of TSPY1 with TSPYL5 on cell proliferation

#### The interaction of TSPY1 with TSPYL5 reinforces cell proliferation in a p53-dependent manner

**Fig. 2:**
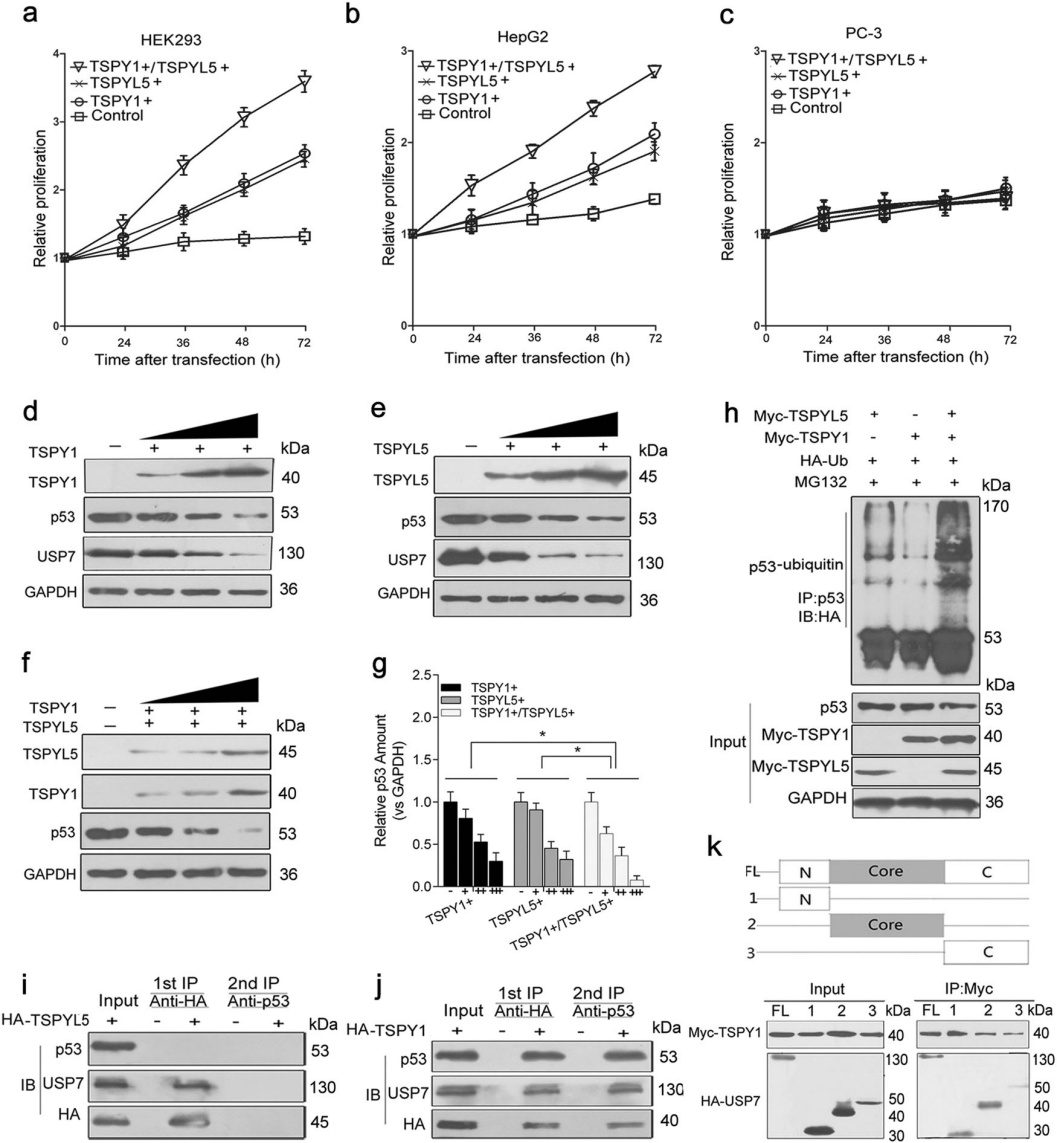
Effect of the interaction of TSPY1 with TSPYL5 on cell proliferation associated with the ubiquitin-mediated degradation of p53. **a**, **b** CCK-8 assays showed that the proliferation levels of p53-expressed HEK293 (**a**) and HepG2 (**b**) cells that overexpressed TSPY1 and TSPYL5 together were significantly higher than those in cells that overexpressed TSPY1 or TSPYL5 alone. Cell proliferation was analyzed at 24, 36, 48, and 72 h after transfection. The total amount of the TSPY1 and TSPYL5 plasmids used for co-transfection was equal to the individual amount of the TSPY1 or TSPYL5 plasmid used when they were transfected separately. The relative proliferation was presented as the fold change, which was calculated based on the absorbance and was normalized to a control value. **c** CCK-8 assays showed that the proliferation of p53-null PC-3 cells was not altered after transfecting the plasmids expressing TSPY1 and/or TSPYL5. **d**–**g** Immunoblotting assays showed that the overexpression of TSPY1 (**d**) or TSPYL5 (**e**) could downregulate p53 level, and the overexpression of both TSPY1 and TSPYL5 (**f**) induced a more significant reduction in the level of p53 in HEK293 cells. Columns (**g**) showing the difference of p53 level in HEK293 cells with different conditions (overexpression of TSPY1 or TSPYL5 vs. overexpression of both proteins). The cells were lysed at 48 h after transfection. Data are presented as the mean ± S.D. (*n* = 3, *p < 0.05). **h** The overexpression of both TSPY1 and TSPYL5 induced a more significant increase in p53 ubiquitylation relative to the overexpression of TSPY1 or TSPYL5 alone in HEK293 cells. The cells were lysed after 10 μM MG132 treatment for 6 h. **i**, **j** Two-step Co-IP assays showed the absence of the TSPYL5-USP7-p53 complex (**i**), while TSPY1, USP7 and p53 could form a complex in HEK293 cells (**j**). **k** Schematic of the USP7 domains and the binding tests of the domains to TSPY1. Co-IP assays showed that TSPY1 could interact with non-p53-specific binding domains of USP7, including the core and C-terminal domains in HEK293 cells

Several studies have reported the acceleration of cell proliferation by TSPY1 and TSPYL5^11,17,18^. Interestingly, we observed that the proliferation level in both HEK293 and HepG2 cells that overexpressed TSPY1 and TSPYL5 together was significantly higher than that in cells that overexpressed TSPY1 or TSPYL5 alone (Fig. 2a, b). Considering that TSPYL5 overrides p53-dependent cell proliferation arrest^18^, we overexpressed TSPY1 and/or TSPYL5 in p53-null PC-3 cells (Supplementary Fig. 2) (ref.^19^). We did not find a substantial difference in cell proliferation between the treatments and control (Fig. 2c). Meanwhile, we overexpressed wild-type p53 and TSPY1 in PC-3 cells and found the suppression of cell proliferation by p53, while TSPY1 could relieve this suppression (Supplementary Fig. 6). Taken together, the results suggest that the interaction between TSPY1 and TSPYL5 reinforces cell proliferation in a p53-dependent manner.

#### The effect of the TSPY1–TSPYL5 interaction on cell proliferation is associated with the ubiquitin-mediated degradation of p53

Downregulation of the p53 protein level was observed in HEK293 cells with TSPY1 or TSPYL5 overexpression (Fig. 2d, e; Supplementary Fig. 7A, B). When the cells were treated with a proteasome inhibitor, MG132, the results indicated that TSPY1 or TSPYL5 could promote 26S proteasome-dependent p53 degradation (Supplementary Fig. 7C, D). A more significant decrease in the p53 level was observed in the cells that overexpressed both TSPY1 and TSPYL5 (Fig. 2f, g), probably due to increased 26S proteasome-dependent p53 degradation (Supplementary Fig. 7E, F). These results suggest that the interaction of TSPY1 with TSPYL5 enhances p53 degradation. In HEK293 cells, we observed that TSPYL5 could competitively bind to the N-terminal domain of deubiquitylase USP7 in conjunction with p53 and reduce the protective effects of USP7 on p53, resulting in ubiquitin-mediated degradation of p53 (Supplementary Fig. 7G-I), and that the co-transfection of TSPY1 and TSPYL5 enhanced this effect (Fig. 2h).

The TSPY1, which is similar to TSPYL5, could increase USP7-mediated p53 ubiquitylation (Supplementary Fig. 7J, K). However, the identification of a complex of TSPY1, USP7, and p53 suggested that unlike the binding of TSPYL5 with USP7, the binding of TSPY1 to USP7 did not exclude the binding of USP7 to p53 (Fig. 2i, j; Supplementary Fig. 7L), which was supported by the finding that TSPY1 could interact with non-p53-specific binding domains of USP7 (Fig. 2k). These findings suggest that there is another mechanism underlying the promotion of ubiquitin-mediated p53 degradation by TSPY1 in addition to that dependent on the TSPY1–TSPYL5 interaction.

#### The interaction between TSPY1 and TSPYL5 promotes cell cycle progression and inhibits cell apoptosis by regulating the activity of p53-target molecules

**Fig. 3:**
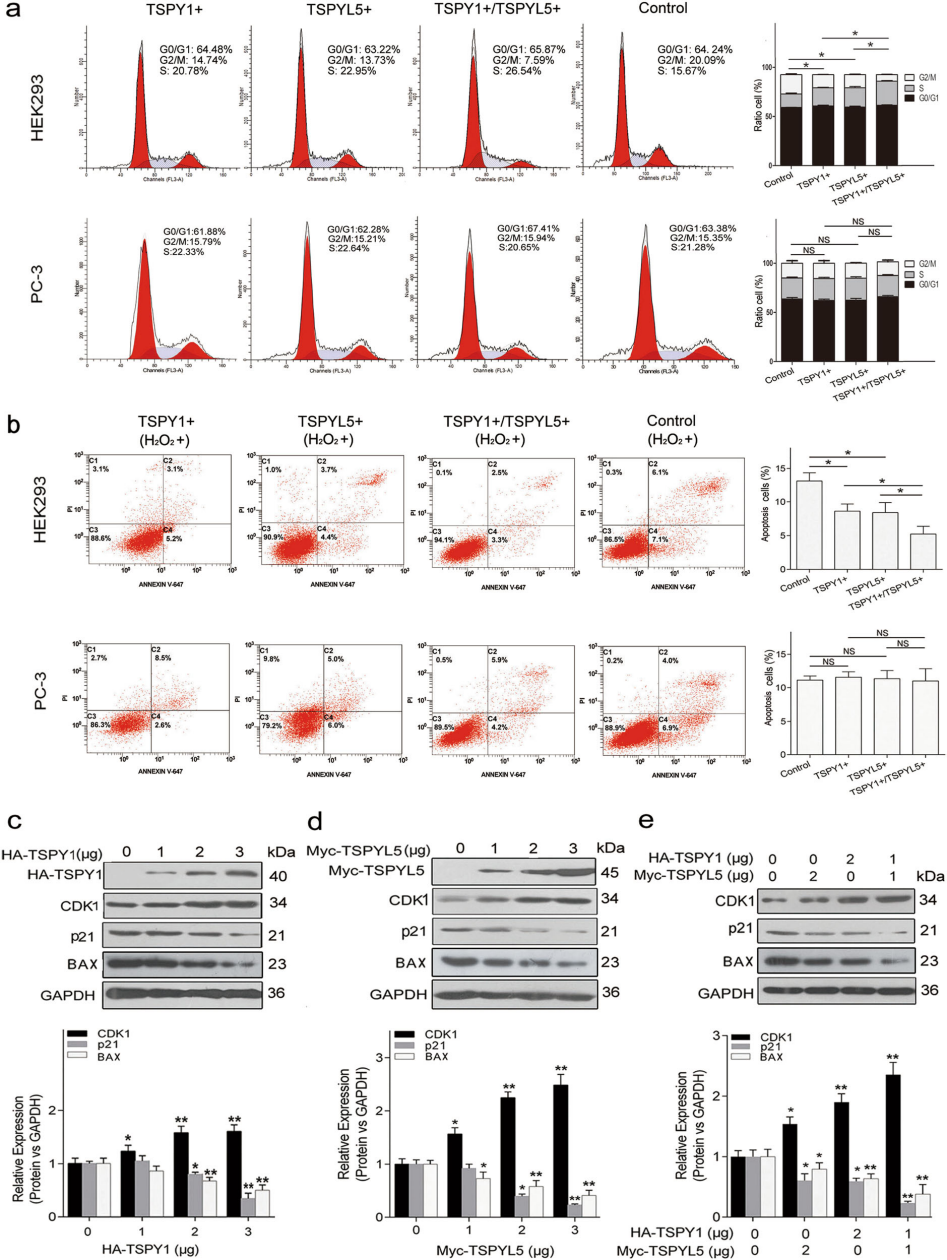
Effect of the interaction between TSPY1 and TSPYL5 on cell cycle progression and apoptosis associated with its regulation of p53-target molecule activity. **a** Cell cycle assays showed a shorter G2/M phase transition in endogenous p53-expressed HEK293 cells that overexpressed TSPY1 and TSPYL5 together relative to those that overexpressed TSPY1 or TSPYL5 alone. The influence of cell cycle by TSPY1 or/and TSPYL5 was absent in p53-null PC-3 cells. The two cells were transfected with TSPY1 or/and TSPYL5 and control plasmids for 48 h and stained with propidium iodide (PI), and the cell cycle was analyzed by flow cytometry. Data represent one of three separate experiments. Columns showing the percentages of G0/G1, S, and G2/M phase cells of HEK293 and PC-3 cells and the alteration in the percentage of G2/M phase population of the two cells after different treatments. The total amount of the TSPY1 and TSPYL5 plasmids used for co-transfection was equal to the individual amount of the TSPY1 or TSPYL5 plasmid used when they were transfected separately. **b** Cell apoptosis assays showed a stronger inhibition of apoptosis in HEK293 cells that overexpressed TSPY1 and TSPYL5 together relative to those that overexpressed TSPY1 or TSPYL5 alone. The influence of cell apoptosis by TSPY1 or/and TSPYL5 was absent in PC-3 cells. After the two cell lines were transfected with TSPY1 or/and TSPYL5 and control plasmids for 48 h, all the cells were treated with 0.1 mM H_2_O_2_ for 16 h. Then, cell apoptosis was analyzed by Annexin V/PI staining and flow cytometry. Data represent one of three separate experiments. Columns showing the percentages of apoptotic HEK293 and PC-3 cells (Annexin V position) and the alteration in the percentage of apoptosis cell after different treatments. **c**–**e** IB assays showed an increase in the CDK1 protein level and reductions in p21 and BAX protein levels in HEK293 cells that overexpressed TSPY1 (**c**) or TSPYL5 (**d**). A more significant increase in the CDK1 protein level and more obvious reductions in p21 and BAX protein levels were observed in HEK293 cells that overexpressed TSPY1 and TSPYL5 together (**e**). The cells were lysed after transfection for 48 h. Columns showing the difference in the levels of CDK1, p21, and BAX in HEK293 with different conditions, including the overexpression of TSPY1 (**c**), TSPYL5 (**d**) and both proteins (**e**). Data are presented as the mean ± S.D. (*n* = 3, *p < 0.05 and **p < 0.01)

Activated p53 can cause cell cycle arrest and induce apoptosis to affect the speed of cell proliferation. We observed a rapid G2/M phase transition in TSPY1- or TSPYL5-overexpressing HEK293 cells, and found a shorter phase transition in cells that overexpressed TSPY1 and TSPYL5 together (Fig. 3a; Supplementary Fig. 8), but a similar alteration was not observed in PC-3 cells (Fig. 3a). Additionally, apoptosis was significantly inhibited in HEK293 cells with increased TSPY1 or TSPYL5 protein levels, and a stronger inhibitory effect on apoptosis was observed in cells that overexpressed both TSPY1 and TSPYL5 (Fig. 3b). However, no inhibitory effect of TSPY1 and TSPYL5 on PC-3 cell apoptosis was found (Fig. 3b). These results indicate that the interaction of TSPY1 with TSPYL5 shortens the cell cycle and inhibits cell apoptosis more significantly than either protein individually through a p53-dependent pathway.

p53 regulates the cell cycle and cell apoptosis via several important target molecules, including CDK1, p21, and BAX^20–22^. Our results showed that the overexpression of TSPY1 or TSPYL5 could promote the transcription of *CDK1* and suppress the transcription of *p21* and *BAX* in HEK293 cells (Supplementary Fig. 9). Additionally, we observed that the reduction in the p53 level was accompanied by an increase in the CDK1 level and a decrease in p21 and BAX levels in the cells that overexpressed TSPY1 or TSPYL5 alone (Fig. 3c, d), with more significant changes in the three protein levels in HEK293 cells that overexpressed TSPY1 and TSPYL5 together (Fig. 3e). These findings suggest that the suppression of p53 function by TSPY1 and TSPYL5 significantly affects the activity of p53-target molecules, further supporting that these two proteins regulate cell proliferation through the p53 pathway.

### Mechanisms of action underlying the regulation of USP7-mediated p53 function by TSPY1

#### TSPY1 enhances the binding capacity of TSPYL5 with USP7

**Fig. 4:**
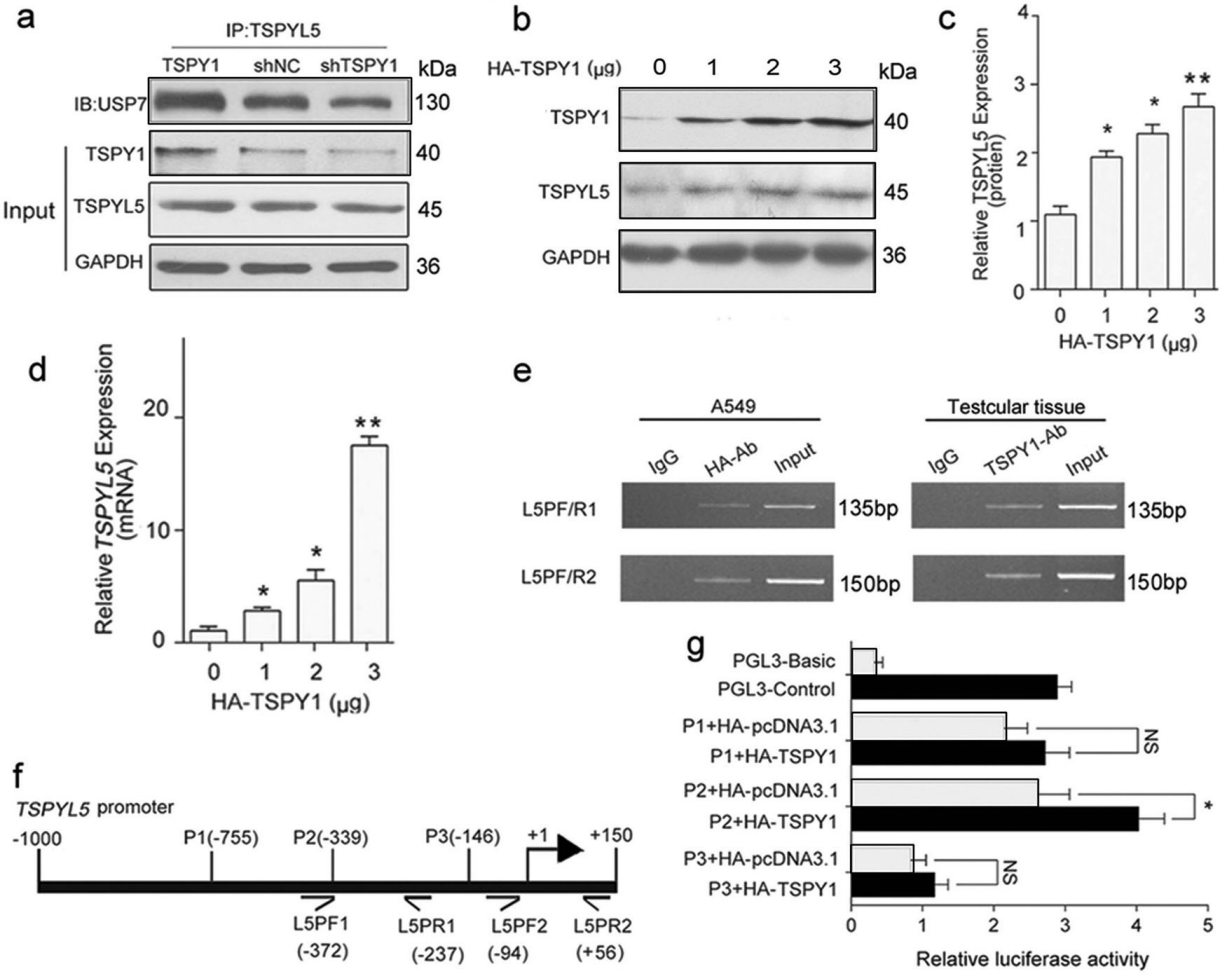
Mechanisms of action underlying the regulation of USP7-mediated p53 function by TSPY1. **a** Co-IP assays showed that more USP7 protein was immunoprecipitated by TSPYL5 in A549 cells that overexpressed TSPY1 than in those with decreased TSPY1 expression. **b**, **c** IB assays showed that overexpressed TSPY1 increased the endogenous TSPYL5 protein level in A549 cells. The cells were lysed after transfection for 48 h. **d** qPCR assays showed a TSPY1 dose-dependent increase in the *TSPYL5* transcription level in A549 cells. Total RNA was extracted from the cells after transfection for 24 h. **e** ChIP-PCR assays of A549 cells and testicular tissues revealed two TSPY1-occupied sites from −372 bp to −237 bp and from -94 bp to +56 bp in the *TSPYL5* promoter. **f** Schematic of the *TSPYL5* promoter depicting the location of ChIP-PCR primers (L5PF1, L5PR1, L5PF2, and L5PR2) and the *TSPYL5* promoter constructs (P1, P2, and P3). **g** Luciferase reporter assays showed that TSPY1 could increase the luciferase activity of the construct with the *TSPYL5* promoter fragment from −755 bp to +150 bp in HEK293 cells. Data are presented as the mean ± S.D. (*n* = 3, *p < 0.05, **p < 0.01). NS not significant

When TSPY1 was overexpressed and knocked down in A549 cells that were co-transfected with TSPYL5, more USP7 was immunoprecipitated by a TSPYL5 antibody in the cells that overexpressed TSPY1 than in the TSPY1-knockdown cells (Fig. 4a). These results indicate that TSPY1 has a beneficial effect on the binding of TSPYL5 to USP7. Because TSPY1, TSPYL5 and USP7 contain the necessary domains to bind to each other in a complex structure, we suggest that TSPY1 and TSPYL5 may bind to different USP7 domains; TSPYL5 interacts with the N-terminus of USP7, while TSPY1 binds to another domain of USP7 to form a stable three protein complex with an increased USP7-anchoring ability relative to the complex formed with TSPYL5 or TSPY1 alone. The structural effect of the TSPY1–TSPYL5 dimer in reinforcing the competitive binding power of USP7 compared with p53 provides an explanation for significantly more p53 degradation in cells that co-overexpress TSPY1 and TSPYL5.

TSPY1 has been suggested to regulate its own expression through a positive feedback mechanism^23^. When overexpressing TSPY1 in A549 cells, we observed an increase in the level of endogenous TSPYL5 in a TSPY1 dose-dependent manner (Fig. 4b, c) and a positive correlation between the TSPY1 level and the abundance of *TSPYL5* mRNA (Fig. 4d), suggesting that TSPY1 can promote the transcription of its homologous gene *TSPYL5*. Furthermore, chromatin immunoprecipitation (ChIP)-PCR assays showed that TSPY1 occupied two sites (from −372 bp to −237 bp and from −94 bp to +56 bp) in the *TSPYL5* promoter (Fig. 4e, f). After constructing three luciferase reporter vectors containing different *TSPYL5* promoter fragments and co-transfecting the constructs with a TSPY1 expression vector individually, we found that TSPY1 could increase the luciferase activity of the construct with a *TSPYL5* promoter fragment from −755 bp to +150 bp (Fig. 4g). These results indicate that TSPY1, by acting as a trans-acting factor, enhances *TSPYL5* gene expression, suggesting that the involvement of TSPY1 reinforces the binding ability of TSPYL5 to USP7 by upregulating the TSPYL5 protein level, which may facilitate USP7-dependent p53 degradation.

#### TSPY1 induces ubiquitin-dependent degradation of USP7

**Fig. 5:**
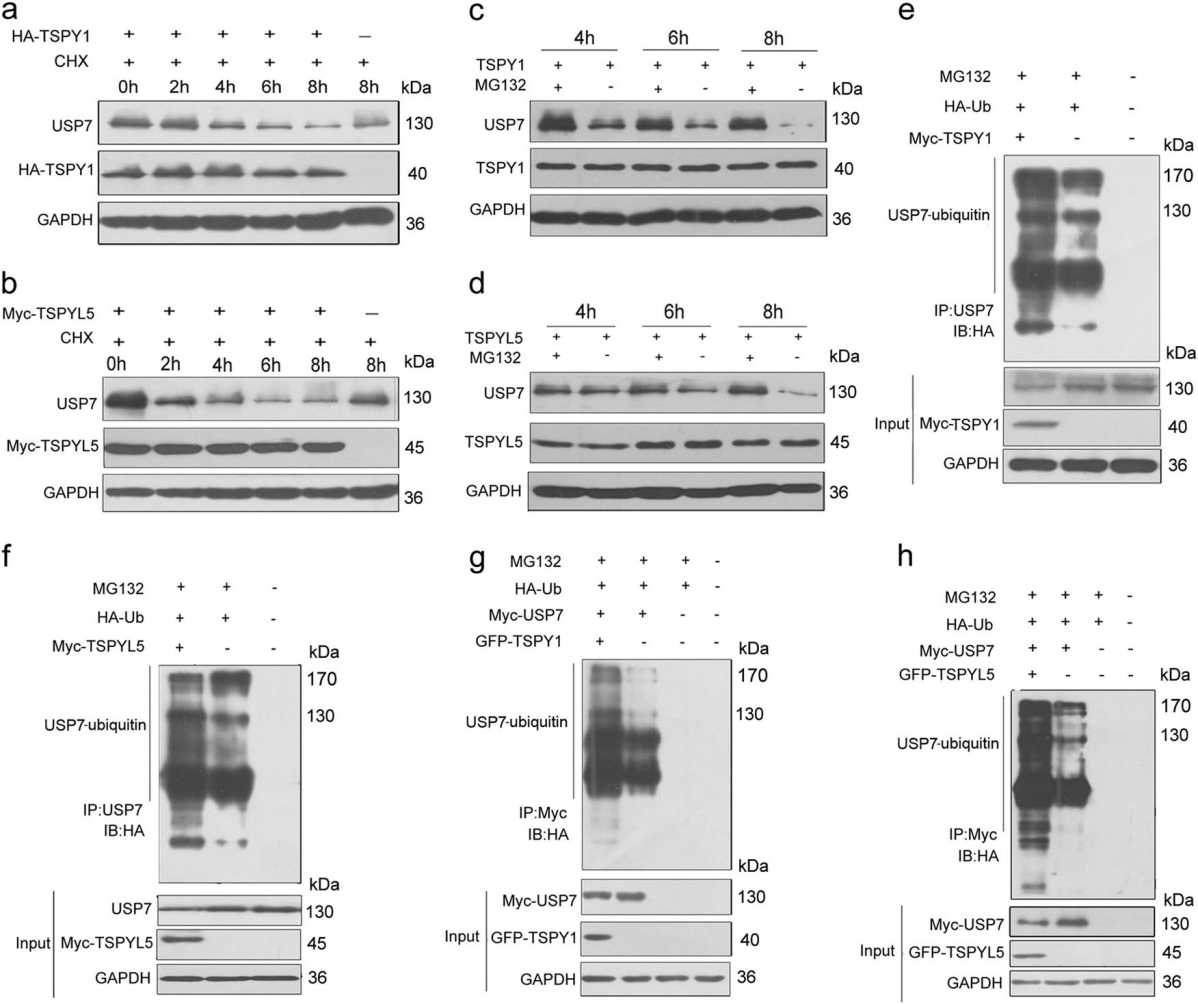
Induction of the ubiquitin-mediated degradation of USP7 by TSPY1 and TSPYL5. **a**, **b** IB assays showed that the overexpression of both TSPY1 (**a**) and TSPYL5 (**b**) induced a notable decrease in the USP7 protein level in HEK293 cells treated by CHX. Cells were treated with 50 μM CHX for 0, 2, 4, 6, or 8 h, respectively, before collection. **c**, **d** IB assays showed that MG132 relieved the reduction in the USP7 protein level in HEK293 cells that overexpressed TSPY1 (**c**) or TSPYL5 (**d**). Cells were treated with 10 μM MG132 treatment for 4, 6, or 8 h, respectively, before collection. **e**, **f** Both TSPY1 (**e**) and TSPYL5 (**f**) increased the ubiquitylation of endogenous USP7 in HEK293 cells. Cells were collected after 10 μM MG132 treatment for 6 h. **g**, **h** Both TSPY1 (**g**) and TSPYL5 (**h**) increased the ubiquitylation of exogenous USP7 in HEK293 cells. Cells were colleced after 10 μM MG132 treatment for 6 h

To explore the mechanism underlying downregulation of the USP7 level by TSPY1 and TSPYL5 (Fig. 2d, e), we treated cells overexpressing TSPY1 and TSPYL5 with a protein biosynthesis inhibitor, cycloheximide (CHX), and observed more obvious degradation of USP7 (Fig. 5a, b); this effect could be inhibited by MG132 treatment (Fig. 5c, d). These observations suggest that TSPY1 and TSPYL5 can induce USP7 degradation via the 26S proteasome system.

Due to the limited influence of TSPY1 and TSPYL5 overexpression on the mRNA level of USP7 (Supplementary Fig. 10A, B), we hypothesized that the functions of TSPY1 and TSPYL5 were involved in the post-transcriptional regulation of USP7 expression. When analyzing their influence on USP7 ubiquitination, we found that the overexpression of TSPY1 or TSPYL5 significantly enhanced the ubiquitination of endogenous and exogenous USP7 (Fig. 5e–h). These findings provide the first evidence that TSPY1, in addition to TSPYL5, can promote the ubiquitin-mediated degradation of USP7, suggesting TSPYL5-independent regulation of USP7-mediated p53 function by TSPY1.

USP7 is a key molecule in the p53 pathway and regulates cell proliferation and apoptosis by stabilizing p53 (ref.^24^). The inductive effect of TSPY1 on the ubiquitination of USP7 prompted a preliminary exploration of the influence of TSPY1 on the expression of thyroid hormone receptor interactor 12 (TRIP12), a newly confirmed E3 ubiquitin ligase targeting USP7 (ref.^25^). Our result indicated the promotion of TRIP12 expression by TSPY1 (Supplementary Fig. 11), providing a clue for investigating the potential mechanism underlying ubiquitin-mediated degradation of USP7 in future studies.

### Impact of TSPY1 on mouse spermatogonia by regulating Usp7-mediated p53 function

**Fig. 6:**
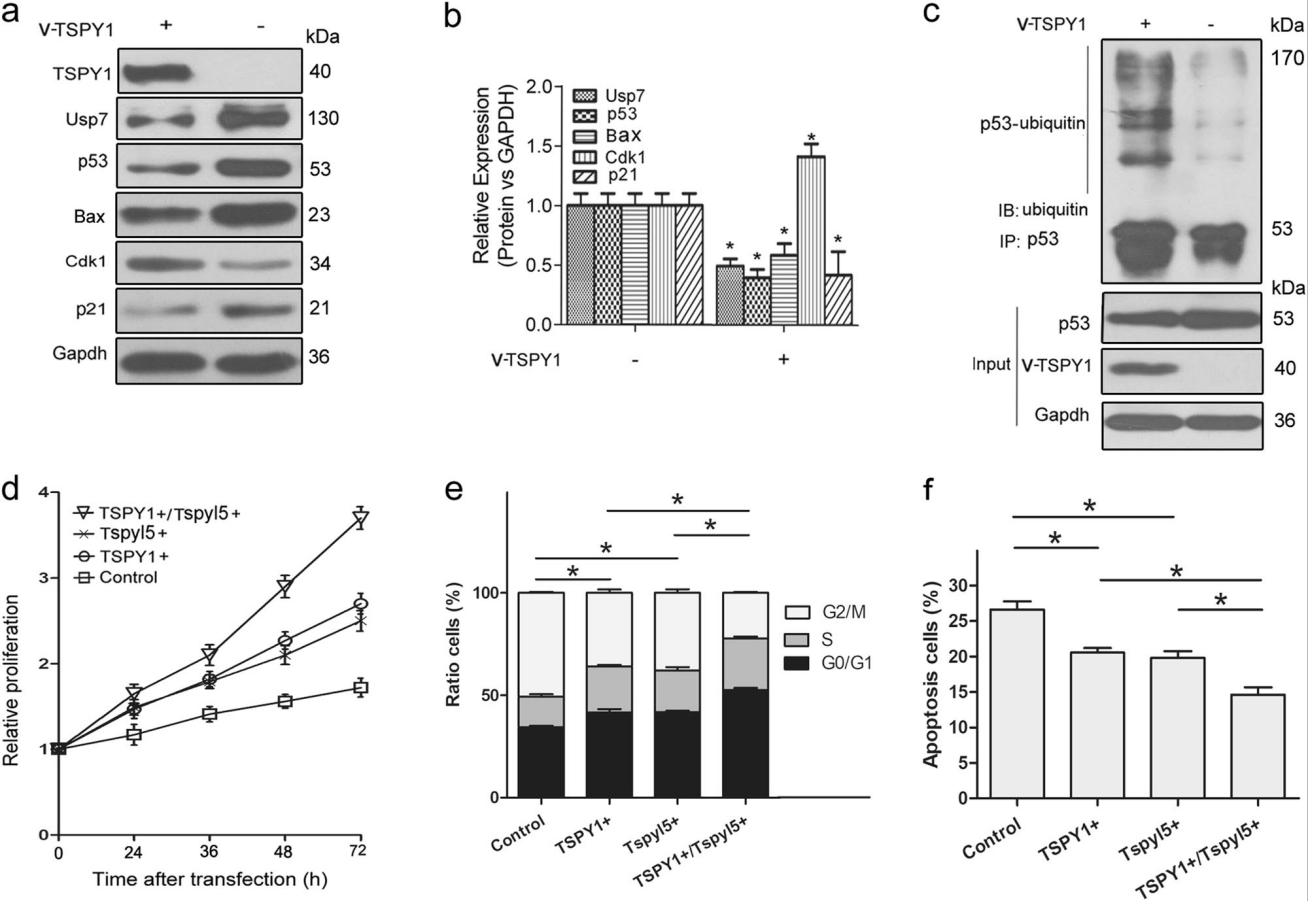
Promotion of mouse spermatogonial proliferation by the interaction of TSPY1 with Tspyl5 via the Usp7-mediated p53 signaling pathway. **a**, **b** IB assays showed that TSPY1 decreased Usp7, p53, p21, and Bax protein levels and increased the Cdk1 protein level in the cells. The cells were lysed after transfection for 48 h. **c** TSPY1 increased the ubiquitylation of p53 in the cells. Cells were treated with 10 μM MG132 for 6 h before collection. **d** CCK-8 assays showed that the overexpression of TSPY1 or Tspyl5 could promote mouse spermatogonial proliferation, and the promotion was more significant in the cells that overexpressed TSPY1 and Tspyl5 together. The relative proliferation was presented as the fold change, which was calculated based on the absorbance and was normalized to a control value. **e**, **f** Cell cycle and apoptosis assays showed that the overexpression of TSPY1 or Tspyl5 could shorten G2/M phase (**e**) and inhibit apoptosis (**f**), and that a more significant effect was present in mouse spermatogonial cells that overexpressed TSPY1 and Tspyl5 together. Columns showing the percentage of G0/G1, S, and G2/M phase cells (**e**) and that of apoptosis cell (**f**), and the alteration in the percentage of G2/M phase and apoptosis cell after different treatments. Data are presented as the mean ± S.D. (*n* = 3, *p < 0.05)

After identifying the binding of human TSPY1 with mouse Tspyl5 in Tspy1-deficient GC-1 cells (Supplementary Fig. 12), we investigated the influence of TSPY1 and Tspyl5 on p53 in cultured spermatogonial cells derived from mouse testes (Supplementary Fig. 13) and observed reduction of the p53 protein level in the cells that overexpressed TSPY1 and/or Tspyl5 (Supplementary Fig. 14). Further analysis showed a reduction in p53, Usp7, p21 and Bax levels and an increase in Cdk1 level in the cells that overexpressed TSPY1 (Fig. 6a, b). A ubiquitylation assay showed that TSPY1 could promote the ubiquitin-mediated degradation of p53 in the spermatogonia cells (Fig. 6c). Furthermore, we observed that proliferation was promoted in the cells that overexpressed TSPY1 and Tspyl5 individually, and increased cell proliferation was found in the cells that overexpressed TSPY1 and Tspyl5 together (Fig. 6d). The overexpression of TSPY1 and/or Tspyl5 could simultaneously shorten the G2/M phase transition of the cells (Fig. 6e) and inhibit cell apoptosis (Fig. 6f; Supplementary Fig. 15). Taken together, these findings indicate that TSPY1 can regulate the cell cycle and apoptosis by suppressing Usp7-mediated p53 function, promoting the proliferation of mouse spermatogonia.

## Discussion

**Fig. 7:**
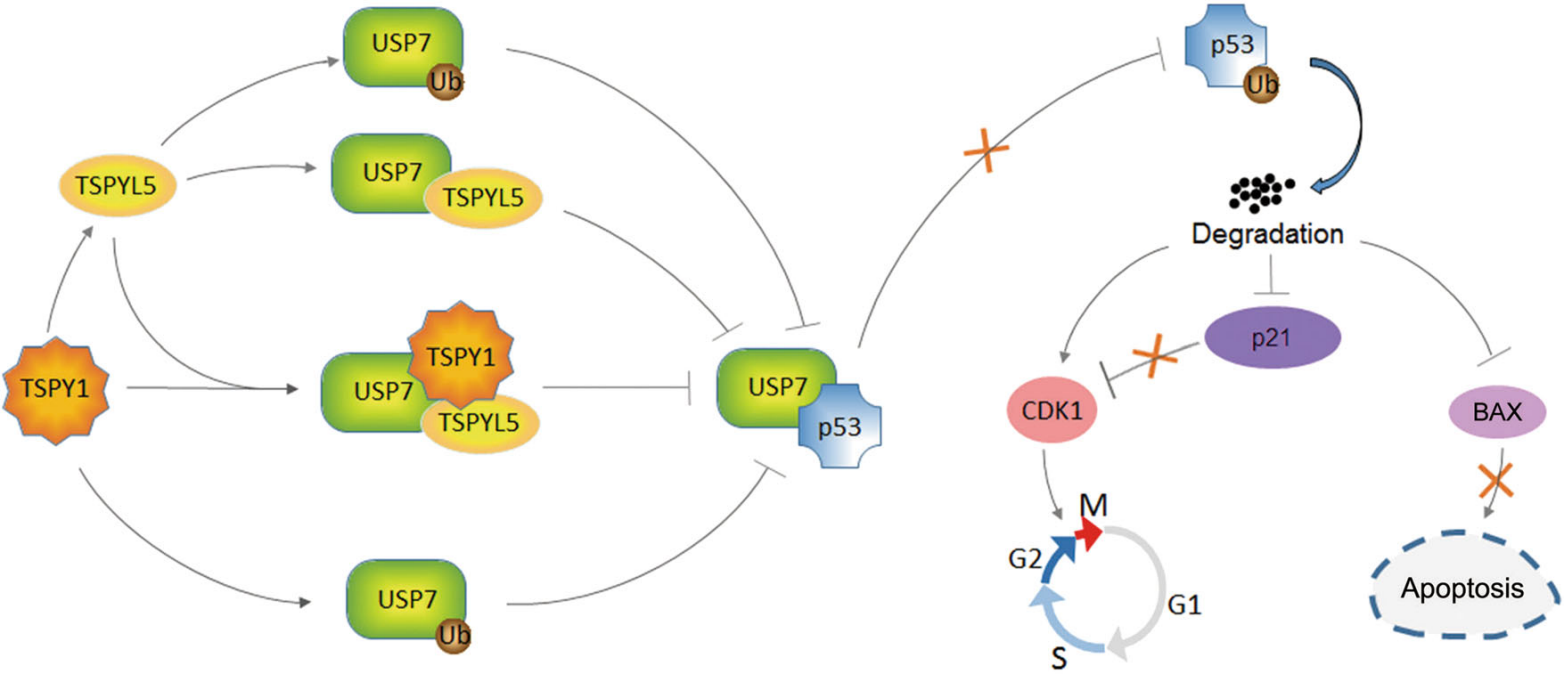
Proposed model for the mechanisms of action underlying the promotion of cell proliferation by TSPY1. TSPY1 promoted USP7-mediated ubiquitin-dependent degradation of p53 by upregulating TSPYL5 expression as a transcription factor, reinforcing the competitive binding of TSPYL5 to USP7 and inducing ubiquitin-mediated degradation of USP7. The suppression of USP7-mediated p53 function by TSPY1 influenced the activity of p53 target molecules (CDK1, p21, and BAX) to expedite the G2/M phase transition and decrease cell apoptosis, accelerating cell proliferation

As a member of the TSPY/TSPYL/SET/NAP (TTSN) protein superfamily, TSPY1 and other TTSN proteins share a highly conserved SET/NAP domain that is involved in DNA replication, transcription modulation, chromatin modeling, and cell cycle regulation^26–29^. Functional studies have revealed several mechanisms of action of TSPY1 underlying its promotion of cell proliferation^11–14^. In this study, we revealed a novel mechanism underlying the promotion of cell proliferation by TSPY1 (Fig. 7). Our findings suggest that TSPY1 forms a complex with TSPYL5 and USP7 to enhance the competitive binding of TSPYL5 to USP7 in conjunction with p53. This phenomenon, together with the promotion of TSPYL5 expression as a transcription factor, impairs the interaction of USP7 and p53 to increase ubiquitin-mediated p53 degradation. Moreover, our experiments showed that TSPY1 could decrease the p53 level by facilitating ubiquitin-mediated USP7 degradation. Our results provide evidence that suppression of p53 function by TSPY1 in a TSPYL5-dependent and -independent manner promotes cell proliferation by accelerating the G2/M phase transition and inhibiting apoptosis.

p53-dependent apoptosis eliminates excessive spermatogonia to maintain germline homeostasis^30–33^. Additional, studies have shown that p53 deletion expands the mouse spermatogonial pool at the expense of spermatogonial self-renewal^30,34^. Conversely, an elevated level of p53 induces excessive apoptosis of spermatogonia and spermatocytes, leading to cell cycle arrest and spermatogenic failure^35,36^. Evidently, these observations suggest that p53 activity must be precisely modulated to maintain normal spermatogenesis. The promotion of mouse spermatogonial proliferation by Tspyl5-mediated p53 signal, strongly suggests that Tspyl5 is involved in mouse germline homeostasis maintenance.

Correspondingly, it is highly possible that the regulation of USP7-mediated p53 function by TSPY1 is an important molecular mechanism underlying its function in human spermatogenesis. First, there is abundant co-expression of TSPY1, TSPYL5 and USP7 in adult human spermatogonia. Notably, when simulating TSPY1 function in Tspy1-deficient spermatogonia cells derived from mouse testes, we observed the promotion of spermatogonial proliferation by TSPY1 along with signaling changes in the Usp7-mediated p53 pathway. These findings strongly support the potential of TSPY1 function in the regulation of human spermatogonial proliferation by suppressing USP7-mediated p53 function. Our previous study showed that males with the common *TSPY1* copy dosages (21~55 copies) have a decreased risk for spermatogenic failure^8^. This observation implies that appropriate TSPY1 dosages, such as those expressed by 21~55 copies of *TSPY1*, may be essential to safeguarding spermatogonial renewal by promoting cell proliferation with a quantal effect; however, TSPY1 dosages determined by extreme copy numbers may dramatically decrease or increase the p53 level and disrupt the homeostasis of the spermatogonial pool, leading to a significant risk for spermatogenic failure.

Although both TSPY1 and TSPYL5 regulate USP7-mediated p53 activity, the influence of TSPY1, compared to TSPYL5, on p53 function may be more dynamic. In our previous study, the *TSPY1* copy dosage was found to exhibit high variability among males^8^; however, our resequencing data from 1558 individuals, including 764 patients with spermatogenic failure and 794 controls with normozoospermia, indicated that the *TSPYL5* sequence was well conserved^37^. These results, together with the upregulation of TSPYL5 expression by TSPY1, suggest that TSPY1 may be a more active regulator of p53 function than TSPYL5 in adult human spermatogonia, highlighting the importance of TSPY1 function in human spermatogenesis. Conversely, abnormal function of the TSPY1 protein under pathological conditions, such as its overexpression in germ cells or ectopic activation in somatic cells, may increase the risk of tumors^38–42^. Our finding that TSPY1 acts as a suppressor of p53 function via the USP7-mediated signaling pathway leads to a novel basic interpretation of the oncogenicity of TSPY1, providing additional insight into the mechanism of action of the cancer/testis protein underlying its promotion of tumorigenesis.

In conclusion, we first demonstrated that TSPY1 suppressed p53 function by reinforcing the competitive binding of TSPYL5 to USP7 in conjunction with p53 and by inducing USP7 ubiquitination. Our finding that TSPY1, as a key component of the testicular p53 pathway, regulates spermatogonial proliferation and apoptosis suggest an important role of the MSY-encoded protein in germline homeostasis maintenance, providing a solid foundation for the diagnosis and treatment of spermatogenic failure and male infertility attributed to TSPY1 deficiency.

## Materials and methods

### Ethical approval

The study, including all experiments involving humans and animals, was approved by the Ethical Review Board of West China Hospital, Sichuan University. Informed consent was obtained from all volunteers who donated testicular tissue samples.

### Human samples

The normal human testicular cDNA library (Cat. #630470) was purchased from Clontech (Mountain View, CA, USA). The testicular tissue samples were obtained from three obstructive azoospermic patients with normal spermatogenesis according to the results of testicular biopsies.

### Human and mouse cells

Human A549 (lung adenocarcinoma), HEK293 (embryonic kidney), HepG2 (hepatocellular carcinoma), PC-3 (prostate cancer), and mouse GC-1 spg (spermatogonia) cell lines were originally purchased from the American Type Culture Collection (Manassas, VA, USA) and then were maintained in our laboratory. The expression conditions of the proteins (TSPY1, TSPYL5, USP7, and p53) in four human cells were evaluated and the results were shown in Supplementary Figure 2. Mouse spermatogonia cells were isolated from the seminiferous tubules of fifteen 8-week-old C57BL/6J male mice using the procedure that was described previously^43^.

### Yeast two-hybrid detection

The full-length cDNA sequence of the *TSPY1* gene encoding 308 amino acids was synthesized and cloned into a yeast two-hybrid bait expression vector pGBKT7 DNA-BD (Clontech). According to the manufacturer’s instructions, the sY187 yeast strain transformed with the bait vector pGBKT7-TSPY1 was mated with the Y2HGold strain containing the normal human testicular cDNA library. After mating, the yeast cells were plated on QD selection media (synthetic drop-out media, ΔLeu, Trp, Ade, His), on which only yeast cells harboring a specific gene sequence encoding a TSPY1-interacted protein could survive. The colonies were tested for α-galactosidase activity on QD/α-galactosidase plates. The selected positive colonies were amplified and propagated in *Escherichia coli* DH5α (Invitrogen, Carlsbad, CA, USA). These positive plasmids, together with either pGBKT7-TSPY1 or an empty pGBKT7 control vector, were retransformed into yeast to verify the interactions. All positive colonies were sequenced, and the corresponding genes were searched using the online BLAST Sequence Analysis Tool (http://blast.ncbi.nlm.nih.gov/Blast.cgi).

### Plasmid construction

The full-length cDNA and partial domain sequences of human TSPY1, TSPYL5, USP7, and p53 and mouse Tspyl5 were synthesized and cloned into a pcDNA^TM^3.1^(+)^ vector (Invitrogen) containing a FLAG, HA or Myc tag sequence. Three *TSPYL5* promoter fragments containing 755 bp (P1), 339 bp (P2), and 146 bp (P3), respectively, were amplified and cloned into the pGL3-Basic luciferase reporter vector (Promega, Madison, WI, USA). Two retroviral vectors overexpressing TSPY1 or Tspyl5 (pEZ-Lv135-TSPY1 and pEZ-Lv122-Tspyl5) were constructed by GeneCopoeia (Rockville, MD, USA). The shRNA designed to interfere with TSPY1 expression and the negative control SuperSilencing shRNA (shNC) were synthesized and cloned into the pGPU6/GFP/Neo vector by GenePharma (Shanghai, China). The target sequence of TSPY1 was 5′-GCTTCTCATTCCACTCCAATT-3′.

### Cell culture and transfection

The primary culture of mouse spermatogonia were performed with a modified procedure based on the protocol described previously^43–45^. Briefly, seminiferous tubules were isolated from testes of fifteen 8-week-old C57BL/6J male mice. The tubules were enzymatically and mechanically dissociated into a heterogeneous cell suspension to generate cultures of testis cells. These cells were plated on laminin to remove the somatic cells, resulting in enriched populations of laminin-binding spermatogonia. The freshly isolated laminin-binding spermatogonia cells were replated on feeder layers of mitomycin C-treated mouse embryonic fibroblasts (MEFs) in DMEM/F12 (Sigma-Aldrich, St. Louis, MO, USA) supplemented with 10% FBS, 20 ng/mL recombinant mouse epidermal growth factor (EGF) (Sigma-Aldrich), 10 ng/mL recombinant human basic fibroblast growth factor (bFGF) (Thermo Fisher, Waltham, MA, USA), 10 ng/mL recombinant rat glial-cell-line-derived neurotrophic factor (GDNF) (Thermo Fisher) and 1000 U/mL murine leukemia inhibitory factor (ESGRO) (Sigma-Aldrich). The constructed retrovirus was transfected into the spermatogonia cells using 5 μg/mL Polybrene (Sigma-Aldrich). Other cells were cultured in DMEM supplemented with 10% FBS at 37 °C in an incubator with 5% CO_2_. The constructed plasmids were transfected into the cells using a jetPRIME transfection kit (Polyplus, Illkirch, France). At 24 and 48 h after transfection, total RNA and protein were extracted from the cells for further quantitative RT-PCR and immunoblotting (IB) analyses.

### Chemical treatments

To evaluate the ubiquitin-mediated degradation of USP7 and p53, the cells were treated with MG132 (Sigma-Aldrich) at a final concentration of 10 μM for 4, 6 or 8 h before they were collected, and the cells were treated with CHX (Sigma-Aldrich) at a final concentration of 50 μM for 0, 2, 4, 6, or 8 h before they were collected.

### Co-IP and IB analyses

The details of the antibodies against the proteins investigated in this study are shown in Supplementary Table S1. Extracted proteins were incubated with 3 µg of target antibodies overnight at 4 °C. Then, the protein A + G agarose beads (40 μl per reaction, Beyotime, Shanghai, China) were added to each incubation sample. The co-immunoprecipitated proteins were separated on 10% SDS-polyacrylamide gels and transferred to a polyvinylidene difluoride (PVDF) membrane (Millipore, Temecula, CA, USA). After blocking in 5% dry milk, the transferred membranes were sequentially incubated with primary antibodies and horseradish peroxidase (HRP)-conjugated secondary antibodies. Then, the immunoreactive bands were identified using a chemiluminescent HRP substrate kit (Millipore) by a molecular imager ChemiDoc XRS + System from Bio-Rad Company (Berkeley, CA, USA). GAPDH was used as the internal reference, and IgG was served as a negative control. The relative protein levels (vs. GAPDH) were calculated using Image J software.

### In vitro binding assays

TSPY1, TSPYL5, USP7, and p53 proteins were individually expressed using the TnT® Quick Coupled In Vitro Transcription/Translation System (Promega). Briefly, 1 μg of each target plasmid DNA was diluted in 50 μl of TNT®Quick Master Mix and incubated at 30 °C for 90 min. Subsequently, 2 μl of each product was used to evaluate the expression of these proteins by IB analysis with specific antibodies. The remaining 30 μl of each translated protein was used for respective binding assays.

### Cell counting kit-8 (CCK-8) assays

A cell counting Kit-8 (Beyotime) was used to analyze cell proliferation. Briefly, 10 μl of CCK-8 solution was added to each well of 96-well plates containing TSPY1 and/or TSPYL5-transfected cells, including HEK293, HepG2, PC-3, and mouse spermatogonia. After 2 h of incubation, the absorbance of each well was measured at 450 nm using a microplate reader. The relative proliferation was determined as the fold change, which was calculated using the absorbance of each well. The results were normalized by the value of a control. Cell proliferation assays were performed in cells transfected with TSPY1 or/and TSPYL5 and control plasmids for 24, 36, 48, and/or 72 h.

### Cell cycle and apoptosis assays

For the cell cycle assay, cells (HEK293, PC-3 and mouse spermatogonia) were fixed with 70% ethanol overnight at 4 °C and treated with RNaseA (0.02 mg/ml) in the dark for 30 min. Then, the cells were stained with propidium iodide (PI, Sigma-Aldrich) and analyzed using a COULTER EPICS XL flow cytometer (Beckman, Krefeld, Germany). Apoptosis was detected using an Annexin V-Alexa Fluor 647/PI apoptosis detection kit (BD Pharmacy, Franklin Lakes, NJ, USA). HEK293 and PC-3 cells were collected and treated with 0.1 mM H_2_O_2_ for 16 h to induce apoptosis. After washing with PBS, the cells were resuspended with 200 μl of 1× binding buffer, to which 10 μl of Annexin V-Alexa Fluor 647 and 5 μl of PI solution was added, and incubated for 15 min at room temperature in the dark. Finally, the apoptosis was analyzed using a COULTER EPICS XL flow cytometer. Cell cycle and apoptosis assays were performed in cells transfected with TSPY1 or/and TSPYL5 and control plasmids for 48 h or 72 h.

### Quantitative PCR (qPCR) assays

Total RNA was extracted using TRIzol reagent (Invitrogen) and was converted to cDNA using a RevertAid First-Strand cDNA Synthesis Kit (Thermo Fisher, Waltham, MA, USA). qPCR was performed using SYBR Premix Ex Taq II (TaKaRa Bio, Dalian, China) on an iCycler RT-PCR Detection System (Bio-Rad). Each assay was performed in triplicate. *GAPDH* was used as a control for human cells, and *Ppp1cc* was used as a control for mouse cells. The primers for qPCRs are listed in Supplementary Table S2.

### Dual-luciferase reporter assays

Three vectors, including pGL3-P1, pGL3-P2, and pGL3-P3, were separately co-transfected with HA-TSPY1 or the negative control pcDNA3.1-HA plasmids into HEK293 cells. After 48 h, the luciferase activity of cell lysates was analyzed using a dual-luciferase reporter assay system (Promega).

### ChIP-PCR assays

ChIP assays were performed using a ChIP-IT Express Enzymatic Kit (Active Motif, Carlsbad, CA, USA). Briefly, the cells were fixed with formaldehyde, and then the cross-linked chromatin was digested using an enzymatic shearing cocktail. A portion of the optimally sheared chromatin was retained as a control “input DNA” in the subsequent PCRs. The remaining chromatin was precipitated by incubation with antibodies against HA and TSPY1. PCR was performed to amplify the target region of the *TSPYL5* gene. The primers for PCRs are listed in Supplementary Table S2.

### Immunohistochemistry

Testicular tissue samples from adult men were fixed with 4% paraformaldehyde overnight, dehydrated in ethanol, embedded in paraffin, and sectioned at 5 μm. For immunohistochemistry, testis sections were deparaffinized, antigen-retrieved at 80 °C in 0.01 M sodium citrate (pH 6.0) for 30 min, treated with 3% H_2_O_2_ for 10 min, and blocked using rabbit serum at 22–24 °C for 1 h. The sections were washed with PBS once and incubated overnight at 4 °C with primary antibodies diluted in PBS (Dilution ratio: TSPY1 1:50 and TSPYL5 1:50). On the next day, the sections were rinsed in PBS three times and incubated for 1 h at room temperature with secondary antibodies. After washing with PBS three times, DAB Chromogen System (DakoCy- tomation, Carpinteria, CA) staining was performed for 5 min and washed with diH_2_O. Finally, sections were counterstained with hematoxylin and stored at 4 °C until analysis. Images were acquired using a laser scanning confocal microscope (Olympus, Tokyo, Japan).

### Immunofluorescence staining

Cells transfected for 24 h were fixed with 4% paraformaldehyde, permeabilized with 0.5% Triton X-100, and blocked with 1% BSA. Then, the cells were sequentially incubated with primary antibodies and DyLight 488- or DyLight 594-labeled secondary antibodies (Invitrogen). To label the cell nuclei, we counterstained the cells with 4, 6-Diamidino-2-Phenylindole, Dihydrochloride (DAPI, Sigma-Aldrich). Images were acquired using a laser scanning confocal microscope (Olympus).

### Terminal deoxynucleotidyl transferase dUTP nick end labeling (TUNEL) assay

Mouse spermatogonia cells on feeder layers of mitomycin C-treated mouse embryonic fibroblasts (MEFs) were permeabilized. Then, the cells were incubated in 50 µl of TUNEL reaction mixture (In Situ Cell Death Detection Kit, Fluorescein, Roche, Indianapolis, IN, USA) for 1 h at 37 °C in a dark and humidified atmosphere. The cell nuclei were counterstained with DAPI. Images were acquired using a laser scanning confocal microscope (Olympus).

### Statistical analysis

The statistical analyses were performed using SPSS 17.0 software (IBM Company, Chicago, IL, USA). Student’s *t*-test was used to compare the observed indexes between the experimental groups. A *p*-value <0.05 was considered significant.

## Electronic supplementary material

Supplementary Figure 1

Supplementary Figure 2

Supplementary Figure 3

Supplementary Figure 4

Supplementary Figure 5

Supplementary Figure 6

Supplementary Figure 7

Supplementary Figure 8

Supplementary Figure 9

Supplementary Figure 10

Supplementary Figure 11

Supplementary Figure 12

Supplementary Figure 13

Supplementary Figure 14

Supplementary Figure 15

Supplementary Tables

Supplementary figure legends
