# Supplementary figures and images for: TSPY1 suppresses USP7-mediated p53 function and promotes spermatogonial proliferation
Source: Cell Death Dis. 2018 May 10;9(5):542. doi: 10.1038/s41419-018-0589-7 (PMC5945610; doi:10.1038/s41419-018-0589-7)

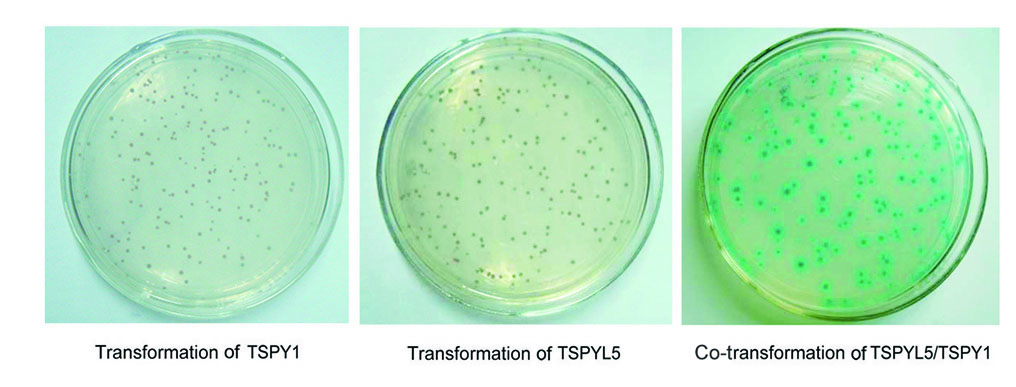

Supplement: Supplementary file 1 — Supplementary Figure 1 [file 41419_2018_589_MOESM1_ESM.jpg]

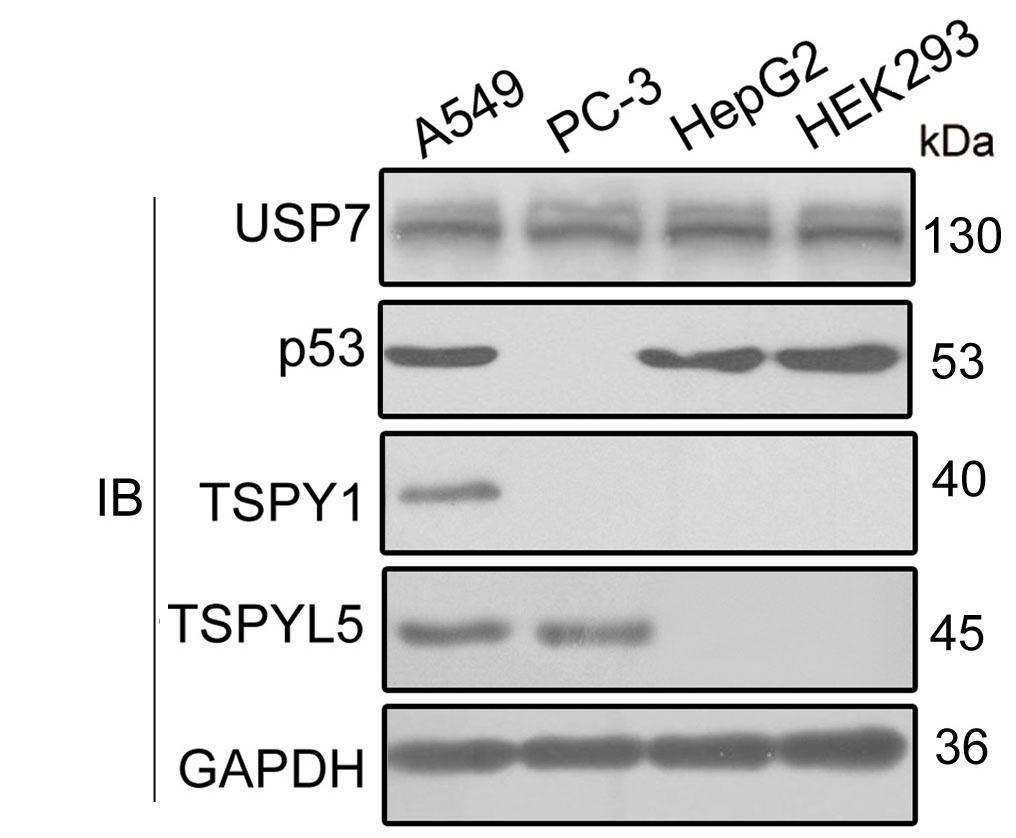

Supplement: Supplementary file 2 — Supplementary Figure 2 [file 41419_2018_589_MOESM2_ESM.jpg]

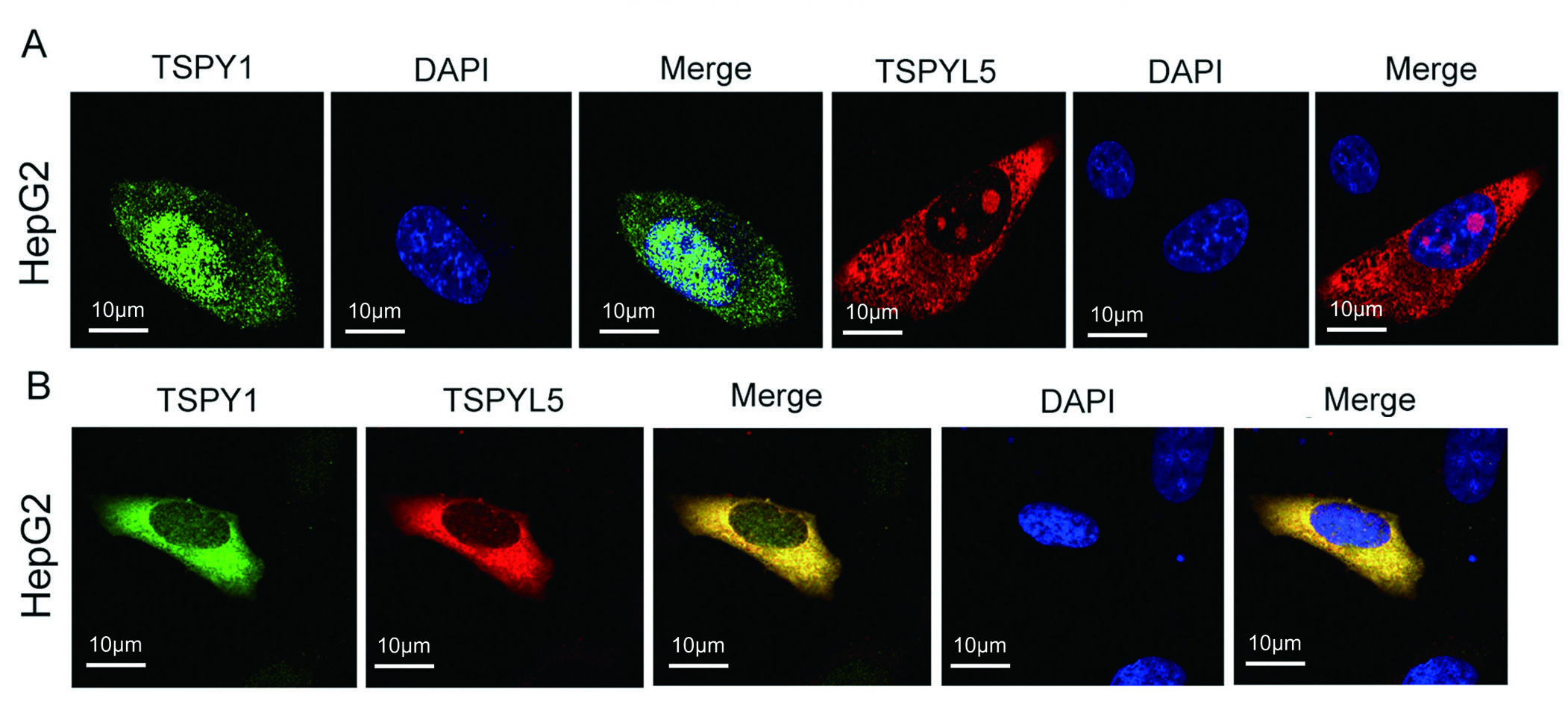

Supplement: Supplementary file 3 — Supplementary Figure 3 [file 41419_2018_589_MOESM3_ESM.jpg]

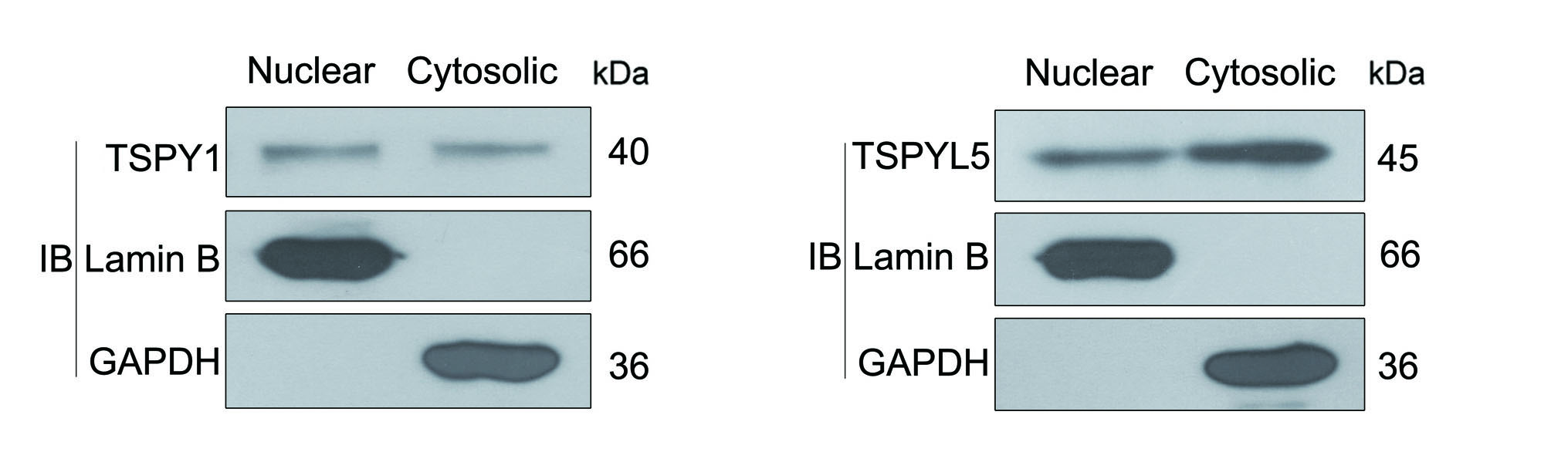

Supplement: Supplementary file 4 — Supplementary Figure 4 [file 41419_2018_589_MOESM4_ESM.jpg]

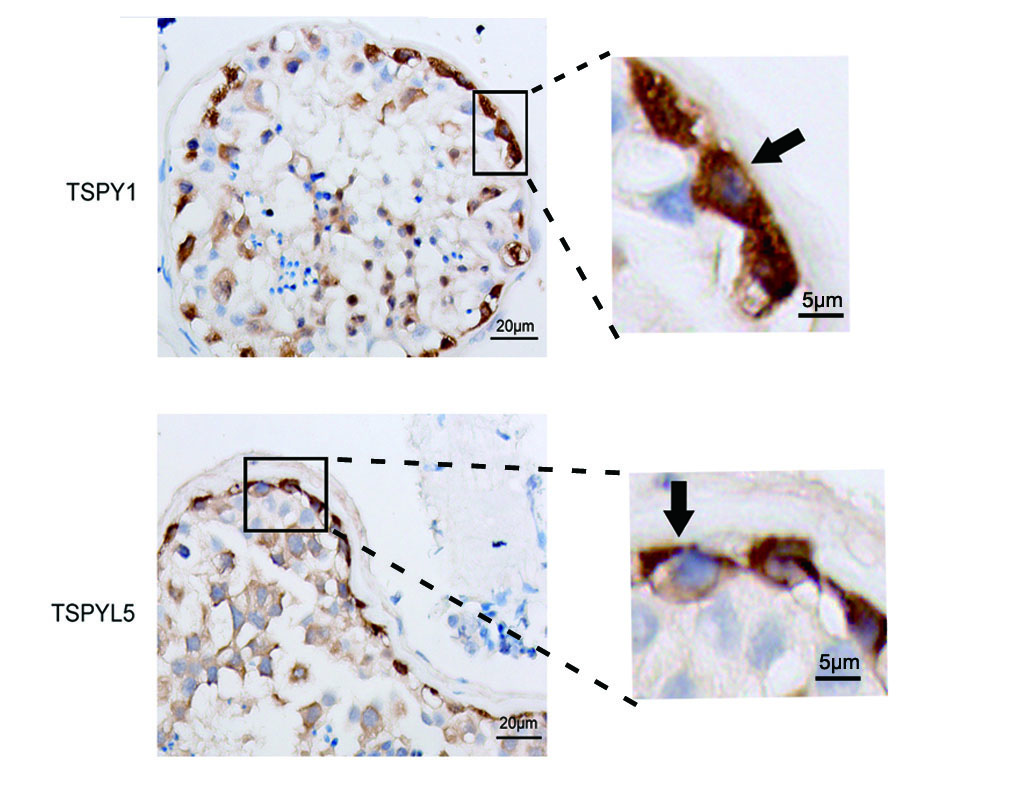

Supplement: Supplementary file 5 — Supplementary Figure 5 [file 41419_2018_589_MOESM5_ESM.jpg]

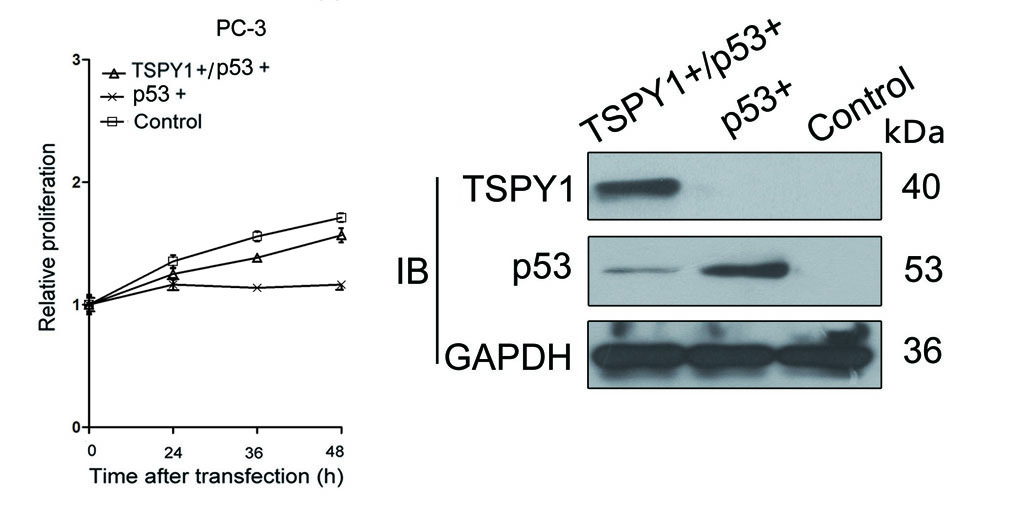

Supplement: Supplementary file 6 — Supplementary Figure 6 [file 41419_2018_589_MOESM6_ESM.jpg]

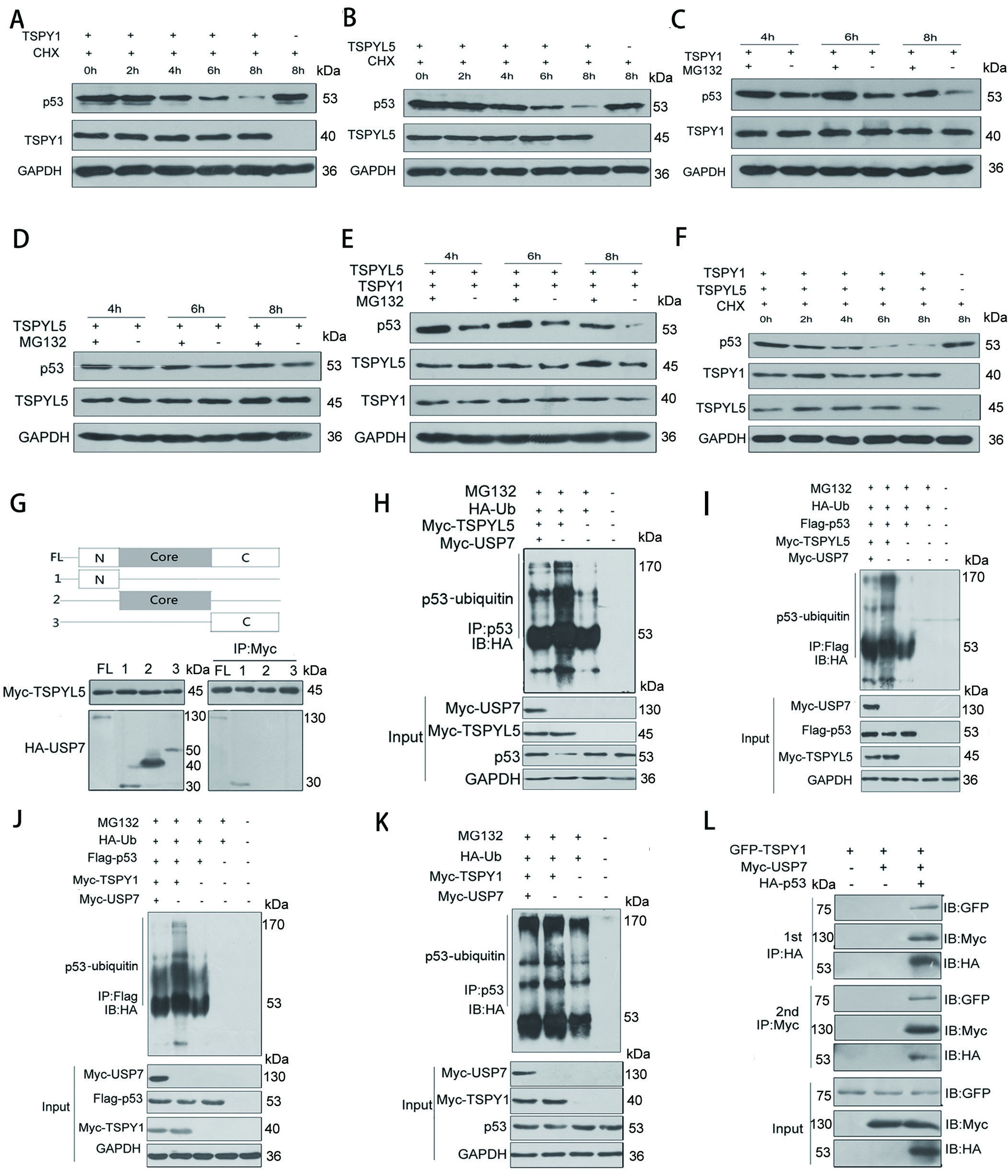

Supplement: Supplementary file 7 — Supplementary Figure 7 [file 41419_2018_589_MOESM7_ESM.jpg]

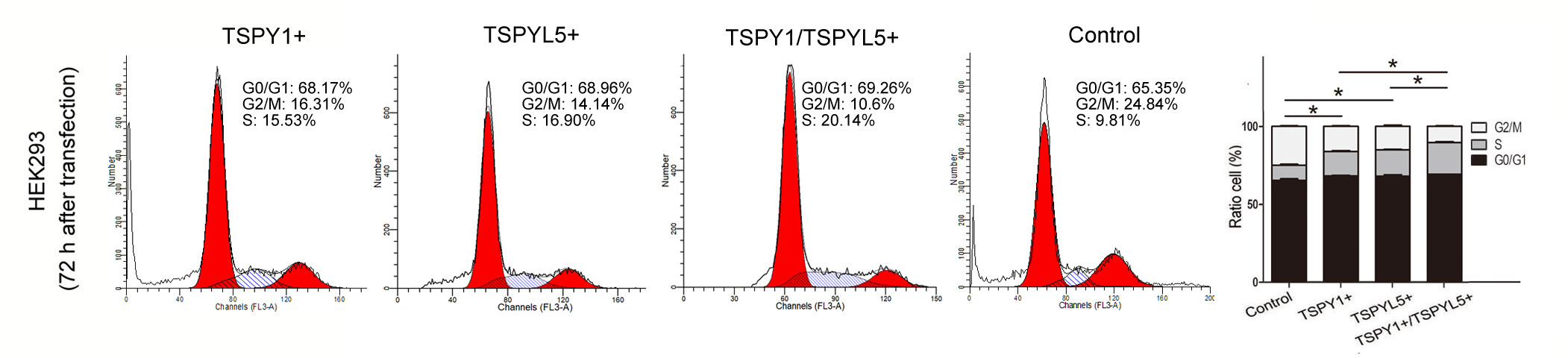

Supplement: Supplementary file 8 — Supplementary Figure 8 [file 41419_2018_589_MOESM8_ESM.jpg]

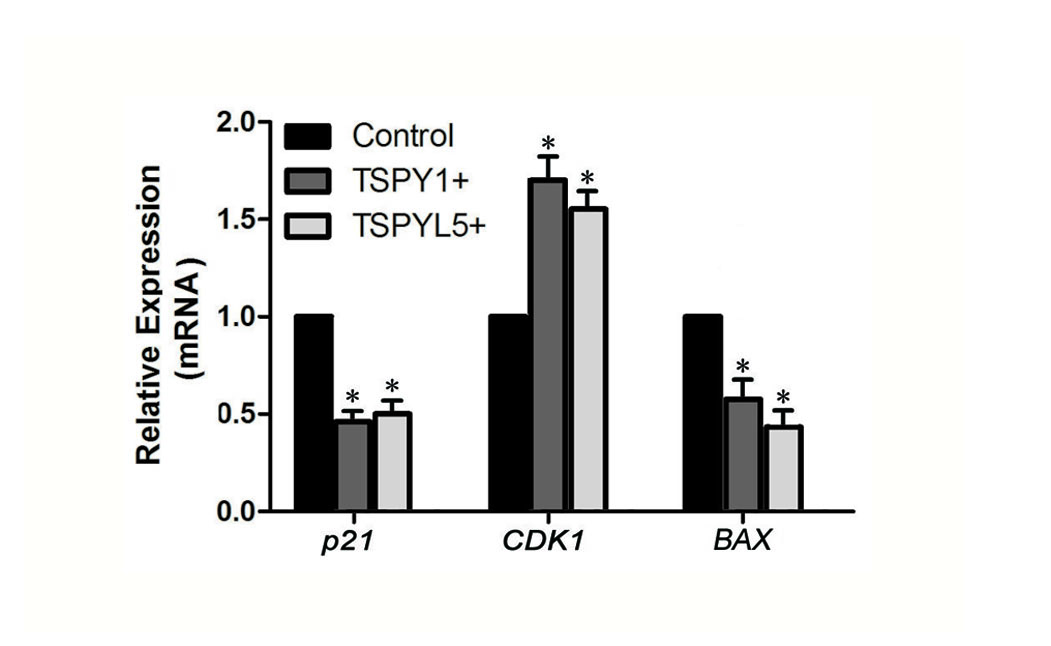

Supplement: Supplementary file 9 — Supplementary Figure 9 [file 41419_2018_589_MOESM9_ESM.jpg]

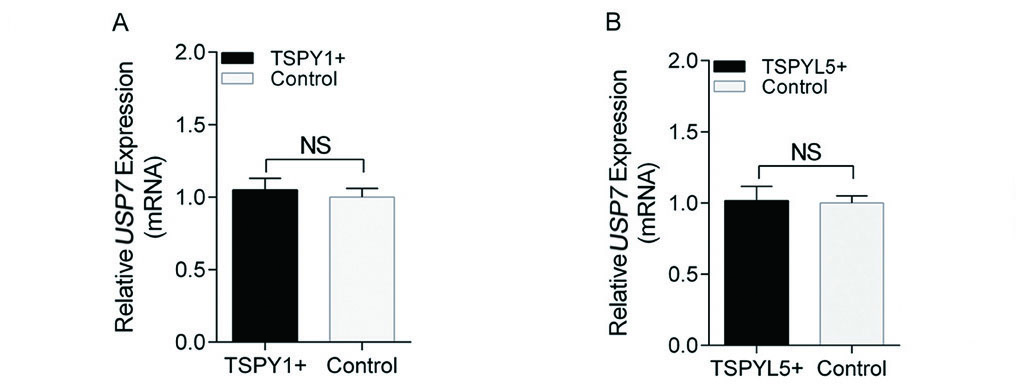

Supplement: Supplementary file 10 — Supplementary Figure 10 [file 41419_2018_589_MOESM10_ESM.jpg]

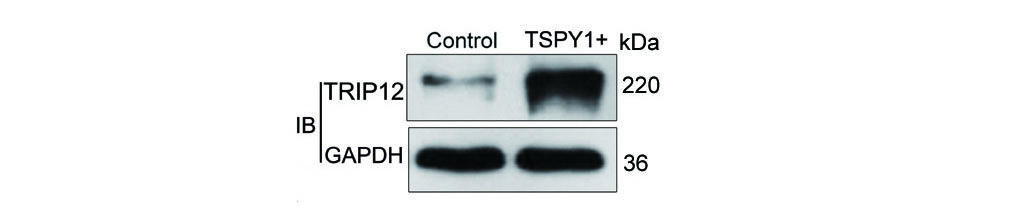

Supplement: Supplementary file 11 — Supplementary Figure 11 [file 41419_2018_589_MOESM11_ESM.jpg]

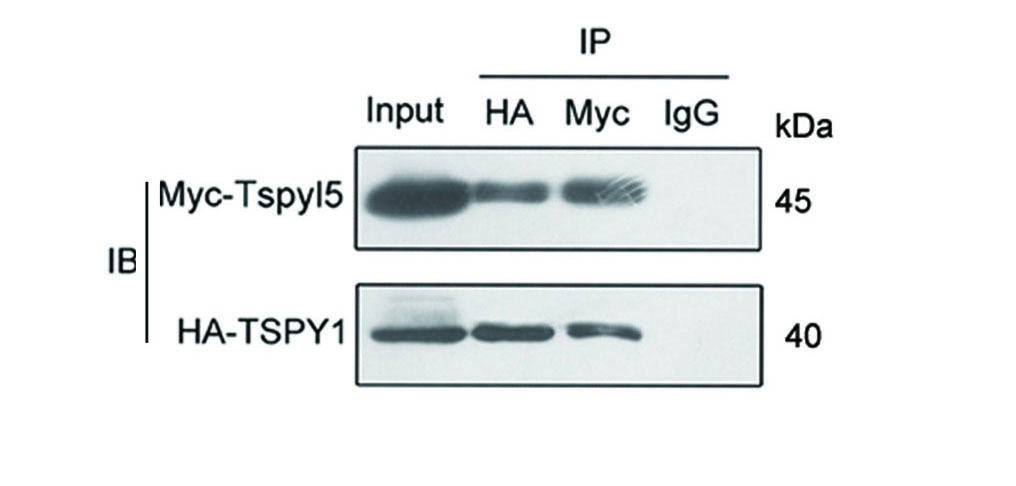

Supplement: Supplementary file 12 — Supplementary Figure 12 [file 41419_2018_589_MOESM12_ESM.jpg]

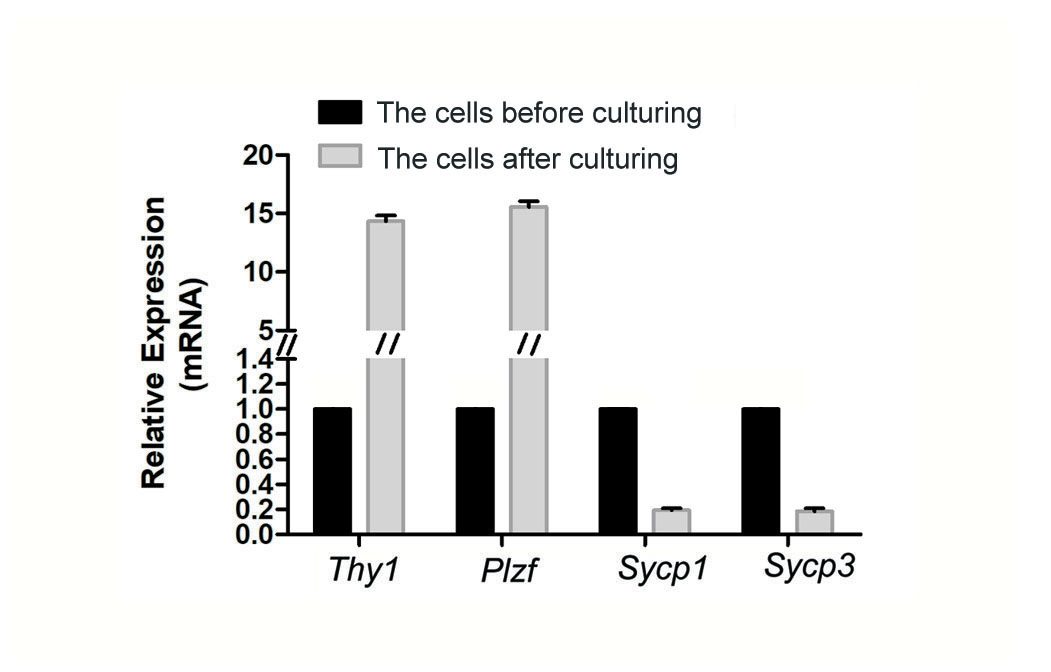

Supplement: Supplementary file 13 — Supplementary Figure 13 [file 41419_2018_589_MOESM13_ESM.jpg]

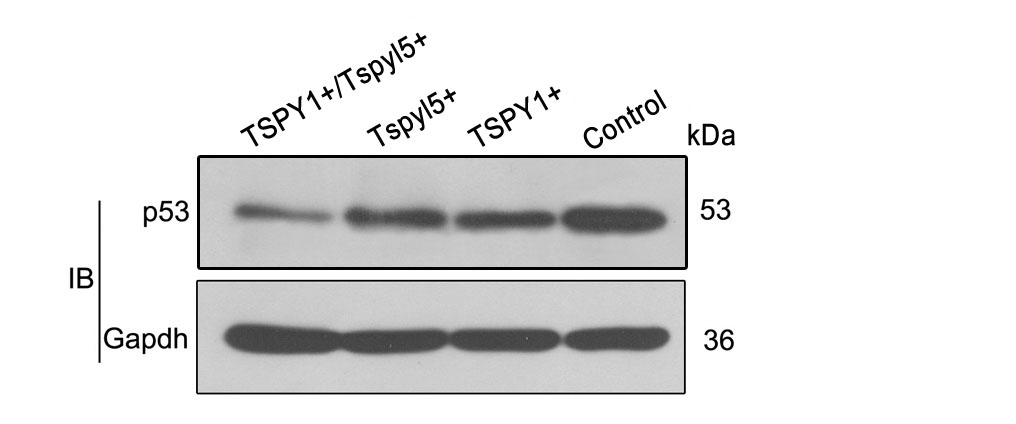

Supplement: Supplementary file 14 — Supplementary Figure 14 [file 41419_2018_589_MOESM14_ESM.jpg]

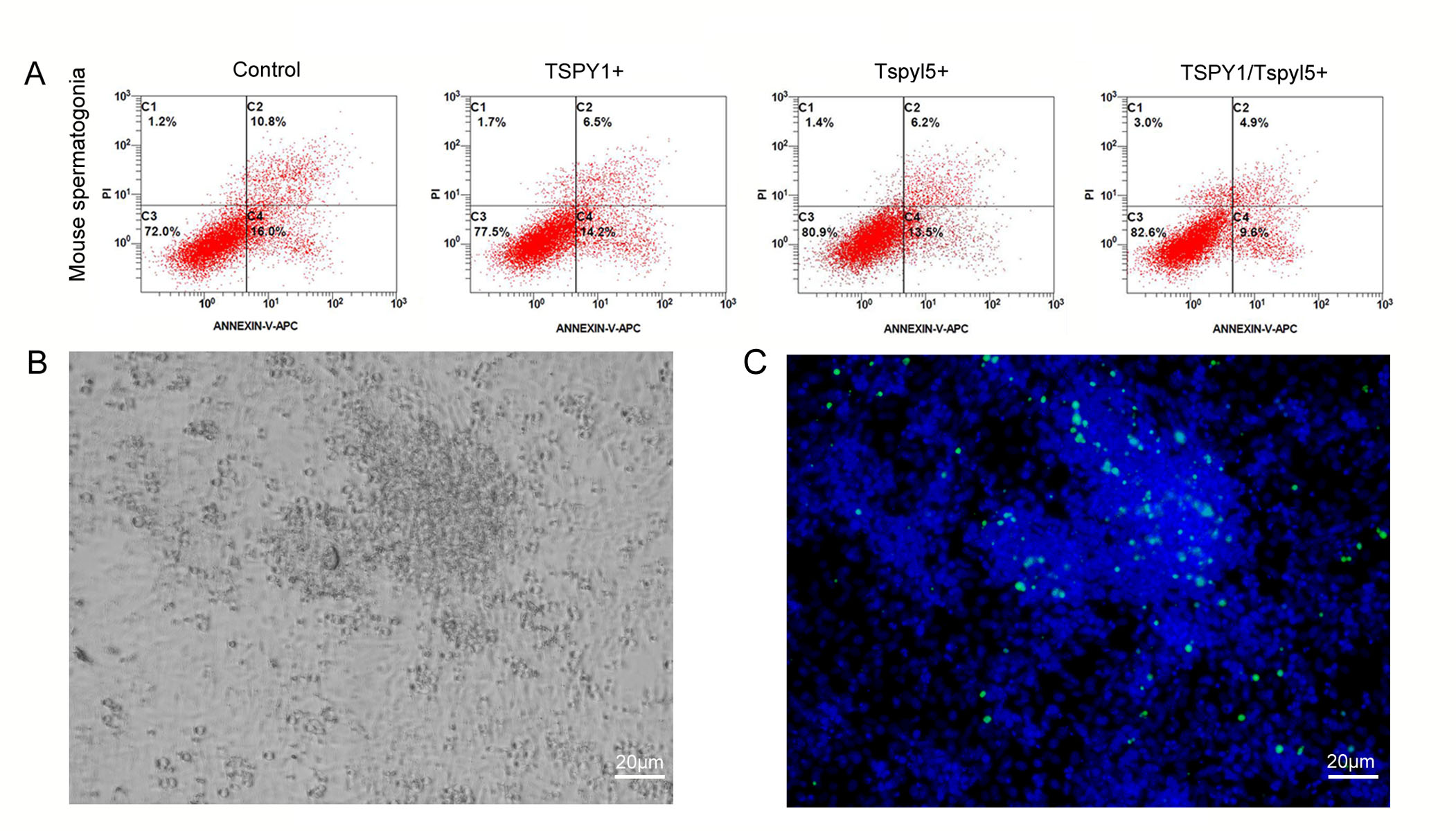

Supplement: Supplementary file 15 — Supplementary Figure 15 [file 41419_2018_589_MOESM15_ESM.jpg]
