# Supplementary material for: TSPY1 suppresses USP7-mediated p53 function and promotes spermatogonial proliferation
Source: Cell Death Dis. 2018 May 10;9(5):542. doi: 10.1038/s41419-018-0589-7 (PMC5945610; doi:10.1038/s41419-018-0589-7)
Supplement: Supplementary file 16 — Supplementary Tables [file 41419_2018_589_MOESM16_ESM.doc]

**Supplementary Table S1**. Details of the antibodies against the proteins investigated in the present study

| **Antibody name** | **Catalogue number** | **Manufacturer** | **Description (Clone number)** | **RRID** |
| --- | --- | --- | --- | --- |
| Anti-TSPYL5 | sc-98186 | Santa Cruz Biotechnology | Rabbit polyclonal (N-15) | AB_2211377 |
| Anti-USP7 | sc-30164 | Santa Cruz Biotechnology | Rabbit polyclonal (H-200) | AB_650256 |
| Anti-p53 for human cell | sc-126 | Santa Cruz Biotechnology | Mouse monoclonal (DO-1) | AB_628082 |
| Anti-p53 for mouse cell | sc-71815 | Santa Cruz Biotechnology | Mouse monoclonal (3H2821) | AB_2209785 |
| Anti-p21 | sc-6246 | Santa Cruz Biotechnology | Mouse monoclonal (F-5) | AB_628073 |
| Anti-CDK1 | ab18 | Abcam | Mouse monoclonal (A17) | AB_2074906 |
| Anti-Bax | sc-20067 | Santa Cruz Biotechnology | Mouse monoclonal (2D2) | AB_626726 |
| Anti-TRIP12 | A301-814A | Bethyl Laboratories | Rabbit polyclonal | AB_1264344 |
| Anti-Ubiquitin | ab7780 | Abcam | Rabbit polyclonal | AB_306069 |
| Anti-Flag | ab49763 | Abcam | Mouse monoclonal (M2) | AB_869428 |
| Anti-Myc | sc-764 | Santa Cruz Biotechnology | Rabbit polyclonal (N_262) | AB_631276 |
| Anti-HA | sc-805 | Santa Cruz Biotechnology | Rabbit polyclonal (Y-11) | AB_631618 |
| Anti-GAPDH | ab8245 | Abcam | Mouse monoclonal (6C5) | AB_2107448 |
| Anti-GFP | 66002-1-Ig | Proteintech | Mouse monoclonal (1E10H7) | AB_11182611 |
| HRP-conjugated goat anti-rabbit | BF03008 | Biodragon |  |  |
| HRP-conjugated goat anti-mouse | BF03001 | Biodragon |  |  |
| DyLight 488-labeled secondary antibodies | A21206 | Invitrogen |  |  |
| DyLight 594-labeled secondary antibodies | A31570 | Invitrogen |  |  |

Mouse monoclonal antibody of TSPY1 is a gift from the laboratory of Prof.Yun-Fai Chris Lau, University of California.

**Supplementary Table S2**. The information regarding the PCR primers used in the present study

| **Target** | **Forward primer（5’—3’）** | **Reverse primer（5’—3’）** | **Product（bp）** |
| --- | --- | --- | --- |
| *TSPY1* | GCTGTTGGATGACATAATGGCG | CCAGGCTGACCATGTAGCTCAG | 368 |
| *TSPYL5* | CGCAGGAACCACCTCATCCA | AATGGAGCTGTGGTTTGAAAAC | 363 |
| *USP7* | CGTTCGGAATCCCGTTTTTGCT | TCAAGGTAAGTGTAGCGACTCC | 280 |
| *CDK1* | AAATGGAAACCAGGAAGC | CATCTTCTTAATCTGATTGTCCA | 165 |
| *p21* | TGAGTTGGGAGGAGGCAG | CGGCGTTTGGAGTGGTA | 207 |
| *BAX* | GGCTGGACATTGGACTTC | CTTCCAGATGGTGAGTGAGG | 156 |
| *GAPDH* | AAGGTCGGAGTCAACGGATTTG | CCTGGAAGATGGTGATGGGATT | 223 |
| *TSPYL5*P1 | AGAAAACAGGTGATGGGGGA | CGAGTTTCTCTGATATGCGCT | 135 |
| *TSPYL5*P2 | GTTGCCGTGGAGAGACCC | CCTTTGCCCCGGTTTTTGG | 150 |
| *Plzf* | GGAGCTGTTCAGCAAGCTG | CCATGTCCGTGCCAGTATG | 320 |
| *Thy1* | TGACCCGAGAGAAGAGGAAG | AGGGCTTGGAGGAGGGAGA | 307 |
| *Sycp1* | ACTCACTCATTTGTGGTGACTG | GGACTTCTTTCTCTCTGGTTTC | 322 |
| *Sycp3* | TAAAGATGGTGCCTGGTGGAA | GCATATTCTGTACTTCACCTCCAAC | 219 |
| *Ppp1cc* | TGTAGAGCCCATCAGGTGGT | TGCTTTGTGATCATACCCCGT | 227 |
