# Supplementary material for: TSPY1 suppresses USP7-mediated p53 function and promotes spermatogonial proliferation
Source: Cell Death Dis. 2018 May 10;9(5):542. doi: 10.1038/s41419-018-0589-7 (PMC5945610; doi:10.1038/s41419-018-0589-7)
Supplement: Supplementary file 17 — Supplementary figure legends [file 41419_2018_589_MOESM17_ESM.doc]

**Supplementary Information**

**Supplementary Fig. 1: Identification of the interaction of TSPY1 with TSPYL5 in AH109 yeast cells.** The transformation of pGBKT7-TSPY1 and pGADT7-TSPYL5 vectors into the AH109 yeast cells individually or together showed that blue clones only grew on selection medium when co-transformed, indicating the physical binding of the two proteins in yeast.

**Supplementary Fig. 2: Detection of TSPY1, TSPYL5, USP7 and p53 protein in human cells.** The endogenous co-expression of p53, TSPY1, TSPYL5 and USP7 was detected in A549 cells. The endogenous USP7 and TSPYL5 were detected while p53 and TSPY1 were absent in PC-3 cells. The endogenous p53 and USP7 were detected while TSPY1 and TSPYL5 were absent in HEK293 and HepG2 cells.

**Supplementary Fig. 3: Locations of exogenous TSPY1 and TSPYL5 in HepG2 cells.** The cells were counterstained with DAPI to label the nuclei (blue). **A** Immunofluorescence assays showed that TSPY1 (green) was mainly located in the nucleus, and TSPYL5 (red) was mainly present in the cytoplasm when the two proteins were individually expressed. The scale bar corresponds to 10 μm. **B** Immunofluorescence assays showed that TSPY1 and TSPYL5 were primarily co-localized in the cytoplasm (yellow) when they were expressed together. The scale bar corresponds to 10 μm.

**Supplementary Fig. 4: Locations of TSPY1 and TSPYL5 in A549 cells.** **A** The limited TSPY1 was expressed with a similar distribution in the cytoplasm and the nucleus. **B** TSPYL5 showed higher expression relative to TSPY1 and was mainly located in the cytoplasm.

**Supplementary Fig. 5: Locations of TSPY1 and TSPYL5 in human testes.** Immunohistochemical staining of human testicular tissue sections showed that TSPY1 and TSPYL5 presented abundant co-expression in the cytoplasm of spermatogonia (black arrows). The scale bar corresponds to 20 μm and 5 μm.

**Supplementary Fig. 6: Influence of TSPY1 and p53 on the** **proliferation in p53-null PC-3 cells.** CCK-8 assays showed that the expression of wild-type p53 could inhibit cell proliferation, and the expression of TSPY1 could rescue the cell proliferation of PC-3 cells that overexpressed wild-type p53. IB assays showed the effectiveness of transfection. The relative proliferation was presented as the fold change, which was calculated based on the absorbance and was normalized to a control value.

**Supplementary Fig. 7: Promotion of the ubiquitin-mediated degradation of p53 by TSPY1 and TSPYL5 in HEK293 cells.** **A, B** The p53 protein level was obviously reduced in CHX-treated cells that overexpressed TSPY1 (A) or TSPYL5 (B). Cells were treated with 50 μM CHX treatment for 0, 2, 4, 6 or 8 h, respectively, before harvest. **C, D** Reduction in the p53 protein level by overexpressed TSPY1 (C) or TSPYL5 (D) could be relieved by MG132. Cells were treated with 10 μM MG132 treatment for 4, 6 or 8 h, respectively, before harvest. **E**, **F** A more significant effect on 26S proteasome-dependent protein degradation of p53 was observed in MG132 (E)- and CHX (F)-treated cells that overexpressed both TSPY1 and TSPYL5 than that in the cells that overexpressed TSPY1 or TSPYL5 alone. **G** Schematic of the three USP7 domains and the binding tests of the domains to TSPYL5. The results showed that TSPYL5 interacted only with the N-terminal domain of USP7 in HEK293 cells. **H** TSPYL5 induced the ubiquitin-mediated degradation of endogenous p53, and USP7 could weaken this effect. Cells were harvested after 10 μM MG132 treatment for 6 h. **I** Exogenous p53 was ubiquitylated when TSPYL5 was overexpressed. Cells were harvested after 10 μM MG132 treatment for 6 h. **J** TSPY1 overexpression increased exogenous p53 ubiquitylation and USP7 could neutralize this effect. Cells were harvested after 10 μM MG132 treatment for 6 h. **K** Endogenous p53 ubiquitylation was enhanced by TSPY1 but reduced by USP7. Cells were harvested after 10 μM MG132 treatment for 6 h. **L** Two-step Co-IP assays showed that TSPY1, USP7 and p53 could form a complex in the *in vitro* TnT system.

**Supplementary Fig. 8: Influence of TSPY1 or/and TSPYL5 on cell cycle in HEK293 cells at 72 h after transfection.** Cell cycle assays showed a shorter G2/M phase transition in HEK293 cells that overexpressed TSPY1 and TSPYL5 together relative to those that overexpressed TSPY1 or TSPYL5 alone. Data represent one of three separate experiments. Columns showing the percentage of G0/G1, S and G2/M phase cells and the alteration in the percentage of G2/M phase after different treatments. Data are presented as the mean ± S.D. (n = 3, **p* < 0.05).

**Supplementary Fig. 9: Regulation of the mRNA levels of the *p21*, *CDK1* and *BAX* gene by TSPY1 and TSPYL5 in HEK293 cells.** TSPY1, TSPYL5 and control plasmids were individually transfected into the cells. The total RNA of HEK293 cells was extracted and *p21*, *CDK1* and *BAX* genes were detected at 24 h after transfection. qPCR results showed that both TSPY1 and TSPYL5 upregulated the the mRNA level of *CDK1* and downregulated those of *p21* and *BAX* in the cells. Data are presented as the mean ± S.D. (n = 3, **p* < 0.05).

**Supplementary Fig. 10: Influence of TSPY1 and TSPYL5 on *USP7* mRNA expression in HEK293 cells.** **A** qPCR results showed that TSPY1 expression did not affect the *USP7* mRNA level. NS, not significant. **B** qPCR results showed that TSPYL5 expression did not affect the *USP7* mRNA level. NS, not significant.

**Supplementary Fig. 11: TSPY1 increased the protein level of TRIP12 in HEK293 cells.**

**Supplementary Fig. 12: Identification of the interaction of human TSPY1 and mouse Tspyl5 in GC-1 cells.** IB assays showed the interaction of human TSPY1 and mouse Tspyl5 in the mouse spermatogonial cells.

**Supplementary Fig. 13: Comparision of the expression level of mouse spermatogonial and spermatocytal markers between the cell groups before and after culturing.** qPCR results showed the more expression amount of spermatogonia markers (*Thy1* and *Plzf*) and less expression amount of spermatocyte markers (*Sycp1* and *Sycp3*) in the cells after culturing relative to the cells before culturing.

**Supplementary Fig. 14: Regulation of p53 level by TSPY1 and Tspyl5 in mouse spermatogonial cells.** IB assays showed that both TSPY1 and Tspyl5 could decrease the p53 level, and the effect was more significant when transfecting TSPY1 and Tspyl5 together.

**Supplementary Fig. 15: Detection of mouse spermatogonial apoptosis.** **A** Cell apoptosis assays showed a stronger inhibitory effect on apoptosis in mouse spermatogonia that overexpressed TSPY1 and Tspyl5 together relative to those that overexpressed TSPY1 or Tspyl5 alone (see Fig. 6f). Data represent one of three separate experiments. **B** The control cells on feeder layers of mitomycin C-treated mouse embryonic fibroblasts (MEFs) were observed by the light microscope. The scale bar corresponds to 20 μm. **C** The TUNEL assay showed the apoptosis of the control cells (green). The scale bar corresponds to 20 μm.

**Supplementary Table S1**. Details of the antibodies against the proteins investigated in the present study

**Supplementary Table S2**. The information regarding the PCR primers used in the present study
